# What do I need to know? Essential educational concepts for complex regional pain syndrome

**DOI:** 10.1002/ejp.1976

**Published:** 2022-06-03

**Authors:** Emily Moore, Felicity A. Braithwaite, Tasha R. Stanton, Valeria Bellan, G. Lorimer Moseley, Carolyn Berryman

**Affiliations:** ^1^ IIMPACT in Health The University of South Australia Adelaide Australia; ^2^ Neuroscience Research Australia Sydney Australia; ^3^ Brain Stimulation, Imaging and Cognition Group, School of Medicine The University of Adelaide Adelaide Australia

## Abstract

**Background:**

Complex Regional Pain Syndrome (CRPS) is a rare but disabling pain condition. Accurate and timely education about CRPS is key to promote optimal clinical outcomes, but it is unclear what the content of that education should be. We aimed to determine the content that both people with CRPS and expert health care professionals (HCPs) reported as important.

**Methods:**

An international three‐round e‐Delphi was conducted, recruiting adults diagnosed with CRPS and HCPs. In Round 1, participants were asked to list the most important information people with CRPS should know regarding the condition*.* Data were organized into concepts and allocated to themes. In Rounds 2 and 3, participants rated each concept on a 9‐point Likert Scale, categorized as ‘not important’ (0–3), ‘important’ (4–6) and ‘very important’ (7–9). A concept attained consensus when ≥75% agreement was reached within a category.

**Results:**

Sixty‐two participants (HCPs: *n* = 7; CRPS: *n* = 55) proposed 193 concepts in Round 1, resulting in 22 themes. Fifteen additional concepts were identified in Round 2, resulting in a total of 208 concepts. From that list, 48 concepts that emphasized understanding and evidence‐based management of the disorder, the importance of self‐management strategies, pacing and movement, reached joint consensus as ‘very important’. One concept: ‘Advise that movement does not help’ reached joint consensus as ‘not important’.

**Conclusion:**

Forty‐eight concepts were jointly considered ‘very important’ for future CRPS‐related educational content. Future research to better understand group differences and to canvas a broader HCP group is warranted.

**Significance:**

This e‐Delphi study identified the 48 core concepts that those with the lived experience of CRPS, and advanced practitioner health care professionals jointly rated as ‘very important’ to include in fundamental and accessible educational material.

## 1 BACKGROUND

Complex Regional Pain Syndrome (CRPS) is characterized by incapacitating pain, as well as sensory, motor, autonomic, bone and skin abnormalities (Goebel et al., 2018; Marinus et al., 2011; Osumi et al., 2018). It affects between 6 and 26 people per 100,000, depending on source data and diagnostic criteria used (Sandorini et al., 2003; de Mos et al., 2007; Elsharydah et al., 2017). CRPS is thought to develop most commonly following soft tissue injuries, fractures or post‐surgery (van Rijn et al., 2011), although it has been reported to arise without any precipitating trauma in up to 9% of cases (Goebel et al., 2012). People with CRPS are often unable to work or effectively carry out activities of daily living, which is associated with lower self‐image (Sweeting et al., 2018), and they have a higher prevalence (15%) of depression than other chronic pain disorders (Brinkers et al., 2018). Over the long term, living with the disorder may result in emotional dysregulation which, along with the presence of depression and severe pain has been linked to suicidal ideation (Jeong et al., 2021; Lee et al., 2014).

Guidelines for optimal management of CRPS support a collaborative partnership between patients and their health care professionals (HCPs). The partnership involves engagement in learning about CRPS, ideally to enable people with CRPS to develop an in‐depth understanding of their condition (Goebel et al., 2019, 2018; Goh et al., 2017; Pardo et al., 2018; Pires et al., 2016). Such a strategy is thought to facilitate critical thinking about the condition and its management, reduce fear of pain (Jong et al., 2005) and promote evidence‐based decisions about management (Pardo et al., 2018; Pires et al., 2016).

People with CRPS, however, often feel like they are facing an unknown enemy, because there is a lack of readily available information about CRPS, and many HCPs lack knowledge of the condition (Grieve et al., 2016; Rodham et al., 2016; Rodham, McCabe, et al., 2012). Furthermore, what readily available information there is, is often inconsistent across sources (Moore et al., 2021) which can lead to feelings of uncertainty about the best way to proceed for the person with CRPS and their HCPs (Grieve et al., 2016). Developing a shared understanding of the disorder is critical for the therapeutic alliance and optimizing outcomes, but people with CRPS find difficulty expressing their experience and HCPs may find it challenging to scientifically explain CRPS (Ashton‐James et al., 2017).

One method to facilitate a shared understanding of the disorder between HCPs and people with CRPS is to develop a consensual concept model using the scientific knowledge of the condition and the lived experience of those diagnosed with it. Consensus may take multiple attempts because of the different perspectives held by HCPs and those living with CRPS (Grieve et al., 2016; Johnston et al., 2015). The Delphi technique (known as an ‘e‐Delphi’, when completed online) provides a structured series of questionnaires to explore fields such as this where potential controversy or lack of clarity exist (Hasson et al., 2000).

Using an e‐Delphi approach, our primary aim was to establish a core set of educational concepts about CRPS, identified and agreed upon by two expert groups (advanced practitioner HCPs and those with CRPS) to guide future educational material.

## 2 METHODS

### 2.1 Research design

To capture an international opinion, an e‐Delphi consensus process was selected. The e‐Delphi is a structured and systematic method of gathering opinions on a topic. Participants are guided to reach a form of consensus by anonymously communicating their opinions, considering the opinions of others and evaluating where their opinions align (Diamond et al., 2014; Fitch et al., 2001; Sinha et al., 2011). The e‐Delphi method is well suited to research in rare conditions because the guide for best practice will come from a relatively small group of experts (Cavero‐Carbonell et al., 2015; Linertova et al., 2012), therefore, large general surveys may be inappropriate.

The e‐Delphi technique has advantages in terms of overall validity when compared with less structured approaches (Sinha et al., 2011). Participants are provided with a platform that allows equal consideration of every opinion, with the additional advantage that participants do not feel obliged to agree with more influential or domineering members (Sinha et al., 2011). This method enables all participants to have an equal voice, regardless of personal circumstances and hence removes bias due to peer pressure, therapeutic interactions and perceived seniority or expertise. The e‐Delphi's online delivery improves accessibility to the survey, enabling participants to conveniently complete it from home or any other internet‐accessible location and to pause and come back/edit their completed surveys an unlimited number of times within the allocated time frame.

### 2.2 Ethical approval, protocol preregistration and reporting guidelines

Ethical approval was granted by the University of South Australia's Human Research Ethics Committee (No. 202185) and the study was preregistered at Open Science Framework (Center for Open Science Charlottesville VA) (https://osf.io/g2tnb; uploaded on 31 January 2020 prior to any analysis taking place). Two deviations from the registered protocol were implemented due to the impact of the COVID‐19 pandemic. To give time for participants to respond whilst coping with local protocols for COVID‐19 management, Round 1 of the survey was extended by a month to a total of 6 weeks and Round 2 by 1 week to a total of 3 weeks.

### 2.3 Participants

Participants from two expert groups were recruited: an advanced practitioner HCP Group (health care professionals/researchers with advanced expertise in CRPS), and a CRPS Group (those living with or recovered from CRPS). See Table 1 for the eligibility criteria for each group.

**TABLE 1:** Participant eligibility and exclusion criteria

Expert group	Eligibility criteria	Exclusion criteria
Living with CRPS	Meets Budapest Criteria requirements to be diagnosed with CRPS, Living/had lived with CRPS for >4 months.	<18 y/o, Failure to meet Budapest Criteria requirements, Onset of symptoms <4 months, Lack of fluency in the English language.
Health care professionals in CRPS management	Members of the European Pain Federation (EFIC) CRPS Taskforce; or Scientific advisors of the Reflex Sympathetic Dystrophy (RSD) Society of America, ≥2 publications on CRPS in 5 years, Expert health background (medicine, physiotherapy, nursing, occupational therapy, or other).	<2 publications on CRPS in 5 years, Publications focused on animal studies or other chronic pain conditions, not specific to CRPS, Lack of fluency in the English language.

The current study aimed to recruit a sample of at least 15 participants per group to increase the likelihood of a representative pool of opinions (Hsu & Sandford, 2007). Because the assessment and management of CRPS are generally poorly understood by health care professionals (Rodham et al., 2016), we were keen to seek the opinions of advanced practitioner HCPs who were experts in the field. We included only recognized and registered professions and extended the inclusion criteria to those who were active in CRPS research and management and had a working knowledge of evidence‐based practice guidelines. The expert HCP group were recruited via email invitation to members of the European Pain Federation (EFIC) CRPS Taskforce and Scientific Advisors of the Reflex Sympathetic Dystrophy (RSD) Society of America to participate in the study. The email contained a link to the first round of surveys, explained the time commitment and importance of the study and included a participant information sheet. Members of the expert Taskforce groups were additionally asked to distribute the participant information sheet to people with CRPS and to CRPS support groups in their area (to promote international representation). Experts living with CRPS were also recruited by the investigators via word‐of‐mouth and past datasets from previously conducted CRPS research. This manner of recruitment ensured confidence that the opinions came from a representative sample with CRPS.

### 2.4 Survey methods and data analyses

Study data were collected and managed using REDCap (Research Electronic Data Capture) electronic data capture tools hosted at the University of South Australia (Harris et al., 2009; Harris et al., 2019). REDCap is a secure, web‐based software platform designed to support data capture for research studies, providing (1) an intuitive interface for validated data capture; (2) audit trails for tracking data manipulation and export procedures; (3) automated export procedures for seamless data downloads to common statistical packages; (4) procedures for data integration and interoperability with external sources.

**FIGURE 1:**
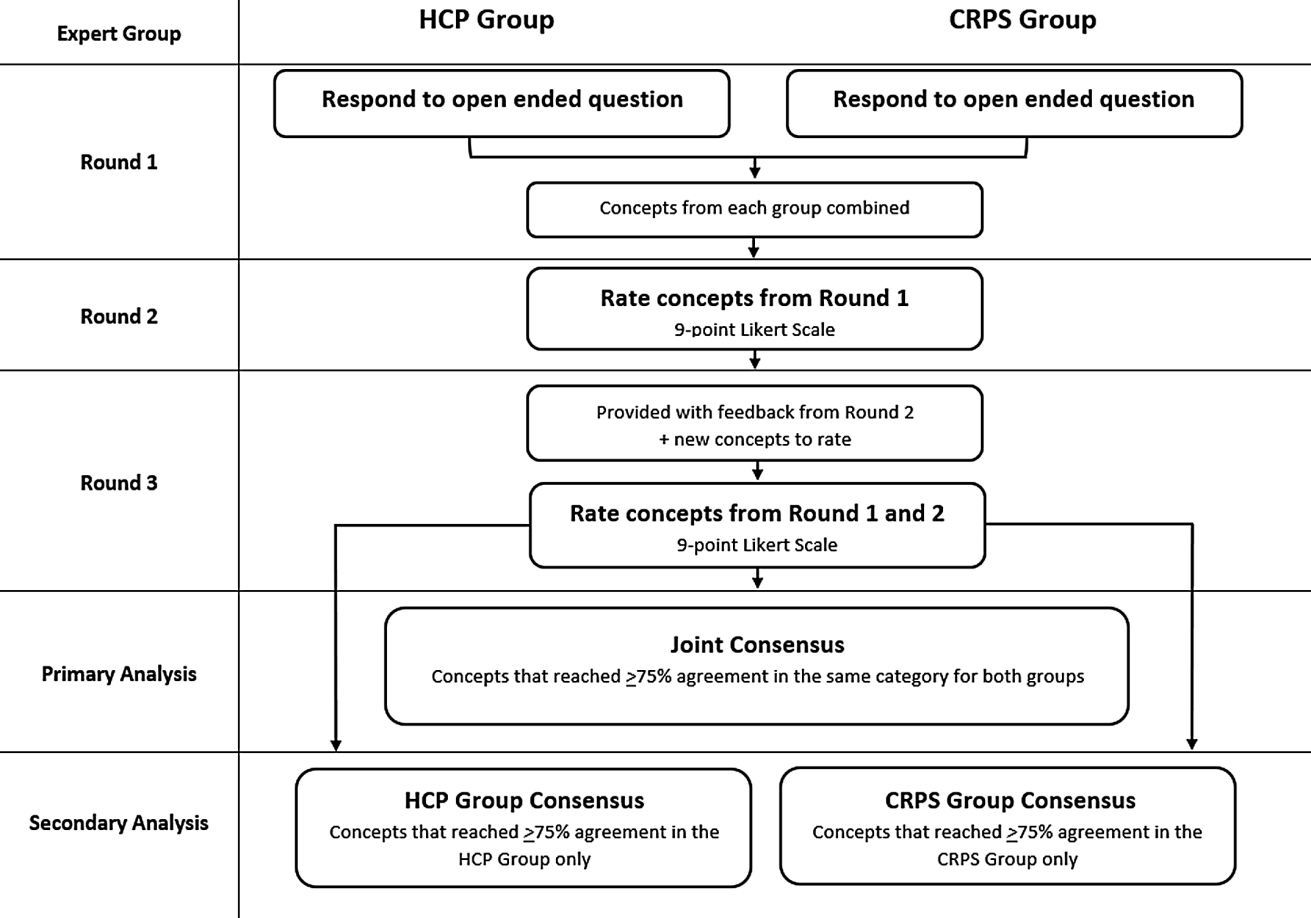
Delphi process.

The e‐Delphi surveys were conducted over an 8‐month period between December 2019 and July 2020. Three survey rounds were conducted (Figure 1).

### 2.5 Pilot testing

Draft versions of all surveys were completed by all the investigators of the study, two independent researchers with REDCap experience, and two individuals living with CRPS to optimize the survey clarity, time commitment and accessibility of the chosen online platform REDCap. Amendments to the surveys were made prior to the commencement of formal data collection. Pilot participants were not eligible to participate in the main study.

#### 2.5.1 Round 1

The survey link for Round 1 opened in December 2019 and closed in February 2020. This round consisted of three sections: (1) study information, including consent processes; (2) responder demographics, and, for participants living with CRPS, lived experiences and IASP Budapest Criteria categories; (3) an invitation to respond to a single open‐ended question: ‘List the most important concepts/information, that you believe people affected by CRPS should know regarding their condition’.

Participants in both groups were additionally asked for their Educational Beliefs—about current CRPS resources and HCP competency. Those with CRPS were also asked how they have educated themselves about the condition and to identify common sources of education. HCPs were asked how they currently diagnose the condition.

Responses from both groups were downloaded verbatim into Microsoft Excel (365) (Microsoft Corporation, 2018) by the primary investigator (EM). These responses were converted into single concepts suitable for a rating on a 9‐point Likert Scale. Two independent reviewers (FAB and CB) placed all related concepts into themes; for example, ‘importance of an anti‐inflammatory diet’ and ‘advice not to eat processed foods’ were placed under the theme ‘Diet’. Theme consistency was reviewed and collated by the primary investigator (EM), and a summary table of themes and concepts was generated and reviewed by the independent reviewers (FAB and CB). Discrepancies about concept placement were resolved by discussion or via consultation with a fourth reviewer (GLM) when disagreement persisted after two reviews. All three reviewers (EM, FAB and CB) assessed each concept for clarity and removal of duplicates.

If appropriate, the original wording provided by participants was retained. However, because English was a second language for many participants, most concepts required some grammatical revisions but retained the original meaning of the words. There were no instances where responses were excluded due investigators to not understanding/interpreting the responses in English effectively. All revisions were evaluated in triplicate (EM, FAB and CB) to maximize the likelihood that the participants' key messages were captured and not skewed by researcher bias. Participants were also provided with the opportunity to clarify or correct any statements, and provide additional information in the next round, a process known as ‘member checking’ (Anney, 2014).

#### 2.5.2 Round 2

In March 2020, the Round 2 survey was sent to all participants who completed Round 1. The Round 2 survey provided a randomized full list of concepts from Round 1. The aim of Round 2 was for participants to consider each concept and evaluate whether it was important to be included in future educational material. Participants were asked to rate each concept on a 9‐point Likert Scale: ‘not important’ (1–3), ‘important’ (4–6) or ‘very important’ (7–9) (Fitch et al., 2001). Participants were also able to comment on each concept, in order to detect potential problems with concepts (e.g. wording/comprehension) and generate new concepts. A priori criteria for consensus agreement required ≥75% of respondents to rate a concept in the same category of importance, consistent with recommendations based on previous Delphi research (Diamond et al., 2014).

#### 2.5.3 Round 3

In June 2020, 2 days prior to Round 3, a feedback summary from Round 2 was provided via email to all participants who completed Round 1. The summary contained information regarding which concepts reached consensus in Round 2 (and, thus, did not require re‐rating), and a list of concepts that did not reach consensus and were to be re‐rated in Round 3 using the 9‐point Likert Scale. Each concept for Round 3 was listed next to a percentage score that highlighted whether a concept was close to reaching consensus (≥75%) in any of the important categories. Participants were asked to consider each concept's percentage score and were given an opportunity to change their previous ratings. Participants were not obligated to change their previous opinions. The feedback was sent 2 days prior for participants to have time to consider the document and their potential changes/responses. Participants were also able to comment on each concept, however, participants were told that any additional information would not be considered as additional concepts at the end of Round 3.

Participants were also notified of the addition of new concepts taken from comment responses in Round 2. New concepts followed the same procedure as responses gathered from Round 1, with comments assessed by EM, FAB and CB, and original wording retained where appropriate.

### 2.6 Statistical analyses

Descriptive statistics were reported for demographic characteristics, IASP Budapest Criteria results, Educational Beliefs, participant response rates and withdrawals. Means, standard deviations and percentage agreement were calculated for each concept based on Round 2 and 3 participant ratings, using Microsoft Excel (365) (Microsoft Corporation, 2018).

#### 2.6.1 Primary analysis

Joint consensus was identified when a concept reached consensus in the same category (e.g. ‘very important’) for both participant groups. Concepts that reached consensus in either the ‘important’ or ‘very important’ categories, for both the participant groups, were considered **essential** to incorporate in future educational material.

#### 2.6.2 Secondary analysis

Group differences in consensus ratings were identified when concepts reached consensus in a category only in one participant group. These were retained for their inclusion in the final recommendations of this study but were not considered for future educational material at this point in time.

## 3 RESULTS

### 3.1 Response rates and demographic characteristics

Sixteen members of the European Pain Federation (EFIC) CRPS Taskforce; or Scientific advisors of the Reflex Sympathetic Dystrophy (RSD) Society of America, were invited by personal email. In turn, they distributed participant information sheets to consumer networks and community distribution boards internationally, along with advertisements on social media sites. Of the 76 experts who volunteered and consented to take part in the current study, 62 participants (*n* = 7 HCPs and *n* = 55 CRPS) completed Round 1 and were invited to Round 2. Sixty‐nine experts living with CRPS consented to participate, however, 13 did not complete the Round 1 survey. Two weeks after providing initial consent, these participants were contacted by the primary investigator (EM) via email, asking them to continue with the Round 1 survey. However, none made further contact and/or attempts to fill in the Round 1 survey. Therefore, these 13 participants were excluded, along with one participant who reported being under the age of 18, resulting in a sample size of 55 in the CRPS Group. Seven individuals within the HCP Group consented to participate and completed the Round 1 survey. Five out of the 7 (71%) HCP participants and 30 of the 55 (55%) experts living with CRPS completed Round 2, leaving an overall sample response rate of 56% for Round 2. For Round 3, 57% of the HCP group and 36% of the CRPS group completed the electronic survey. Table 2 presents within‐survey response rates.

**TABLE 2:** Within‐survey response rates and exclusions

	Response rates × responded/consented (proportion)		
	Professionals (*n =* 7 consented)	Individuals living with CRPS (*n =* 69 consented)	Overall (*n =* 76 consented)
Round 1	7/7 (100%)	55/69 (80%) −14 excluded	62/76 (82%)
Round 2	5/7 (71%)	30/55 (55%)	35/62 (56%)
Round 3	4/7 (57%)	20/55 (36%)	24/62 (39%)

### 3.2 Group demographics

All experts living with CRPS met the symptom‐based threshold (i.e. none were physically assessed, making verification of signs impossible) for the IASP Budapest Diagnostic Criteria for CRPS (Harden et al., 2010). Participants reported having continuing pain (100%), sensory changes (98%), swelling changes of their affected limb (91%), excess sweating (70%), colour changes (96%), skin, hair or nail changes (89%), temperature regulation changes (98%) and movement/control changes (96%). More detailed reports about the lived experience are provided in Table S1. Demographic information for the CRPS and HCP Groups is presented in Table 3.

**TABLE 3:** Expert group demographic characteristics

Characteristic	HCP group (*n =* 7)	CRPS group (*n =* 55)
Age (years, mean [SD])	52 (6.36)	45 (13.2)
Age range (years)	41–59	24–72
Gender (F:M)	2:5	50:5
Country born (count)	Germany (3) Denmark (1) Switzerland (1) The Netherlands (1) United States (1)	The Netherlands (29) Canada (16) Australia (2) Belgium (2) Germany (2) Jamaica (1) Korea (1) South Africa (1) United States (1)
Country currently living	Germany (2) Denmark (1) Switzerland (1) The Netherlands (1) United Kingdom (1) United States (1)	The Netherlands (29) Canada (19) Australia (2) Belgium (2) Germany (2) Bali (1) France (1) Korea (1) United States (1)
HCP group		
Highest Educational Qualification	Post‐Graduate degree (7)	
Approx. how many CRPS patients would be seen a year	0–3: 1 20–30: 1 40–50: 2 50 and over: 3	
Current role	Professor or Associate Professor: 5 Head of Clinical Unit/Department: 2	
Clinical background	Physician: 5 Neurologist: 2	
CRPS group		
No. of participants currently Living with CRPS	52	
No. of participants recovered from CRPS	3	
Amount of time recovered from CRPS	5–12 months: 2 10 years and over: 1	
CRPS duration (recovered and current)	4–12 months:36 1–2 years: 12 3–5 years: 5 10 years and over: 1	
Areas affected by CRPS symptoms including pain (recovered and current)	Just upper limbs: 19 Just lower limbs: 23 Both upper and lower limbs affected: 7 ^a^Trunk: 1 ^b^Whole body: 6	

^a^ Trunk refers to CRPS signs or symptoms reported in body parts other than the limbs.

^b^ Whole‐body refers to CRPS signs and symptoms reported in all limbs or trunk, as defined above.

### 3.3 Educational belief results

The majority of participants in both the CRPS group (34/55) and the HCP group (4/7) reported that they believed people with CRPS have access to adequate resources regarding CRPS. Of the CRPS group (47/55) reported that they educated themselves on CRPS via the Internet, and (30/55) sought information from online CRPS support groups. The majority of both groups (CRPS: 32/55, HCP: 5/7), however, reported that they believed HCPs, in general, are not up‐to‐date on CRPS information. Only 22/55 experts living with CRPS believed that they received adequate information about CRPS from their health practitioner. See Tables S1 and S1 for further information on Education Beliefs and how people with CRPS sought information about their condition, respectively. Table S1 provides lived experience reports.

### 3.4 Concepts and themes derived from the e‐Delphi surveys

#### 3.4.1 Round 1 and round 2 results

A total of 208 concepts (Round 1 = 193 concepts; Round 2 = 15 concepts) emerged from the e‐Delphi survey process and were allocated into 22 themes.

### 3.5 Round 3 results

#### 3.5.1 Primary analysis

**FIGURE 2:**
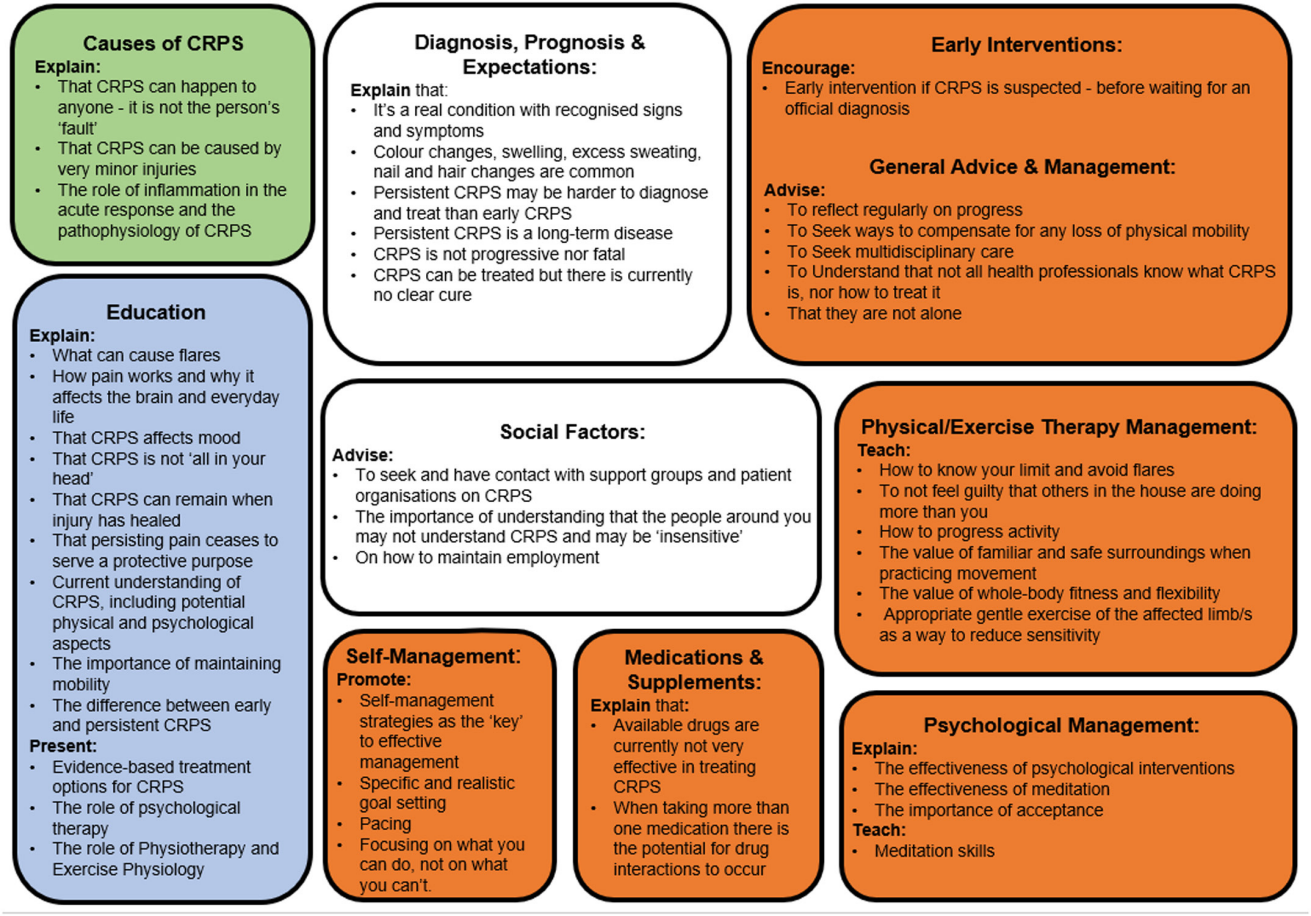
Visual representation of concepts that shared a ‘very important’ rating in both the CRPS and HCP groups independently. Green = causes, blue = understanding/education, white = diagnosis and social factors and Orange = management strategies.

Joint consensus was reached for 49 concepts. Within the 49 concepts, 48 reached consensus as ‘very important’ and one as ‘not important’. The 48 ‘very important’ concepts are summarized in Table 5 and visually represented in Figure 2, with Table S1 providing detailed results. The single concept that was considered ‘not important*’* was: ‘Movement does not help’ [81% consensus, mean = 2.2 (*SD* = 1.67)]. Of the 22 themes, 15 contained concepts that reached joint consensus and the theme with the highest number of concepts (*n* = 8) was ‘Management strategies—Physiotherapy, Exercise and Pacing’.

#### 3.5.2 Secondary analysis—Conceptual differences between the groups

On average, participants in the HCP group rated each concept lower in importance [mean = 5.9 (*SD* = 1.96)] than participants in the CRPS group [mean = 7.3 (*SD* = 1.66)].

The CRPS group reached consensus on 80 concepts (in addition to the 49 discussed above), rating all 80 as ‘very important’. Of the 80 concepts that reached consensus, the top 5 concepts were: (I) ‘CRPS does not define you; it's just a part of your life. You are more than your pain, more than your disability’ [100% consensus, mean = 8.5 (*SD* = 0.73)]; (II) ‘It is important to get as much rest and sleep as possible. Try to get at least 8 h of sleep each night’ [100%, mean = 8.3 (*SD* = 0.88)]; (III) ‘It is important to have good sleep hygiene’ [100%, mean = 8.4 (*SD* = 0.76)]; (IV) ‘Your medical support team needs to understand CRPS and you need to be happy with the team’ [100%, mean = 8.4 (*SD* = 0.82)]; (V) ‘Information to explain CRPS to your friends and family is necessary so they understand your pain and limitations’ [96%, mean = 8.4 (*SD* = 0.84)].

The HCP group reached consensus on 32 concepts (in addition to the 49 above), rating 18 as ‘very important’, four as ‘important’ and nine as ‘not important’. Of the 18 concepts that reached consensus as ‘very important’, the top 5 concepts were: (I) ‘Persistent CRPS is unlikely to resolve quickly’ [100%, mean = 8, (*SD* = 1.15)]; (II) ‘CRPS is an exaggerated response to an accident, injury, surgery or trauma’ [100%, mean = 7.8 (*SD* = 0.74)]; (III)’It can be very distressing for people with CRPS to feel like their limb does not belong to them’ [75%, mean = 7.8 (*SD* = 1.5)]; (IV) ‘It is important to address any psychosocial issues associated with CRPS by seeking the advice of a pain psychologist’ [100%, mean = 7.8, (*SD* = 0.5)]; (V) ‘There is generally a good prognosis with early CRPS' [100%, mean = 7.6, (*SD* = 0.8)].

The four concepts that reached consensus as ‘important’ for the HCP group were: (I) ‘CRPS symptoms and pain can change along with the changes in weather’ [80% consensus; mean = 4.6 (*SD* = 1.49)]; (II) ‘It is normal to go through the stages of grief for the losses you may experience with CRPS’ [80%; mean = 4.6 (*SD* = 1.49)]; (III) ‘It's OK to rest and put your feet up to help the symptoms’ [75%; mean = 6.3 (*SD* = 2.06)]; (IV) ‘Those living with CRPS may benefit from tracking their pain and symptoms’ [75%; mean = 6.3 (*SD* = 2.06)].

Of the nine concepts that reached consensus as ‘not important’ for the HCP group, the top 5 concepts were: (I) ‘Drink at least 2 litres of water per day’ [100% consensus; mean = 1.8 (*SD* = 0.95)]; (II) “Chaga Mushrooms are effective in managing CRPS' [75%; mean = 2.3 (*SD* = 1.25)]; (III) “Salty sea water is an effective natural remedy’ [75%; mean = 2.5 (*SD* = 1.29)]; (IV) ‘Diet can be helpful in managing CRPS' [75%; mean = 2.5 (*SD* = 1.29)]; (V)‘Diet is effective in managing CRPS' [7.5%, mean = 2.8, (*SD* = 1.70)].

The CRPS group reached the highest average importance rating level for the Stress theme [mean = 8.1 (*SD* = 1.22)], with the Alternative Therapies theme having the lowest average importance rating [mean = 5.9 (*SD* = 2.39)]. For the HCP group, the Phenotype theme reached the highest average importance rating level [mean = 7.5 (*SD* = 1.73)], with the Diet theme having the lowest average importance rating [mean = 2.8 (*SD* = 1.78)]. See Table 4 for Theme results.

**TABLE 4:** Theme and concept ratings across the groups

	Combined participant groups (HCP + CRPS)		HCP group		CRPS group	
Theme (*n* = no. of concepts within theme)	Average rating (M [SD])	No. of concepts that reached consensus (not important: Important: Very important)	Average rating (M [SD])	No. of concepts that reached consensus (not important: Important: Very important)	Average rating (M [SD])	No. of concepts that reached consensus (not important: Important: Very important)
Phenotype (n = 1)	7.1 (1.73)	0: 0: 0	7.5 (1.73)	0: 0: 1	7.1 (1.76)	0: 0: 0
Diagnosis (*n* = 4)	7.3 (1.78)	0: 0: 3	6.7 (2.29)	0: 0: 1	7.4 (1.65)	0: 0: 3
Prognosis (*n* = 8)	6.8 (2.00)	0: 0: 4	7.2 (1.29)	0: 0: 6	6.8 (2.08)	0: 0: 3
Mechanism—“Not all in the head” (*n* = 6)	7.6 (1.42)	0: 0: 5	7.1 (1.34)	0: 0: 6	7.7 (1.37)	0: 0: 5
Mechanism—causes (*n* = 10)	7.3 (1.66)	0: 0: 7	6.9 (1.89)	0: 0: 7	7.4 (1.57)	0: 0: 6
Lived experience (*n* = 20)	7.3 (1.90)	0: 0: 12	6.0 (2.07)	0: 1: 5	7.6 (1.64)	0: 0: 14
Social factors/support (*n* = 13)	7.3 (1.68)	0: 0: 9	6.1 (1.67)	0: 1: 4	7.6 (1.54)	0: 0: 10
Education/understanding (*n* = 17)	7.3 (1.71)	0: 0: 13	6.3 (1.63)	0: 0: 7	7.5 (1.59)	0: 0: 12
Individualisation (*n* = 4)	7.5 (1.93)	0: 0: 4	5.2 (2.26)	0: 0: 0	8.0 (1.4)	0: 0: 4
Medications/supplements (*n* = 13)	6.5 (2.01)	0: 0: 3	5.8 (2.01)	0: 0: 2	6.7 (1.98)	0: 0: 9
Alternative therapies (*n* = 4)	5.5 (2.5)	0: 0: 0	3.4 (1.67)	2: 0: 0	5.9 (2.39)	0: 0: 0
Hydrotherapy (*n* = 4)	6.6 (2.25)	0: 0: 0	4.7 (3.5)	0: 0: 0	7.0 (1.78)	0: 0: 0
Stress (*n* = 1)	7.7 (1.63)	0: 0: 1	6 (2.09)	0: 0: 0	8.1 (1.22)	0: 0: 1
Diet (*n* = 7)	5.6 (2.57)	0: 0: 0	2.8 (1.78)	5: 0: 0	6.3 (2.26)	0: 0: 0
Early intervention (*n* = 7)	7.2 (1.75)	0: 0: 4	5.9 (1.73)	0: 0: 2	7.5 (1.62)	0: 0: 4
Health care advice (*n* = 13)	7.6 (1.65)	0: 0: 10	6.5 (1.70)	0: 0: 6	7.8 (1.44)	0: 0: 10
Self‐management and advocacy (*n* = 11)	7.3 (1.74)	0: 0: 7	6.3 (2.19)	0: 1: 4	7.6 (1.50)	0: 0: 7
Management strategies—psychological (*n* = 9)	7.5 (1.85)	0: 0: 8	6.4 (2.16)	0: 0: 3	7.7 (1.64)	0: 0: 8
Management strategies—physiotherapy, exercise and pacing (*n* = 20)	7.0 (1.82)	1: 0: 15	5.9 (2.35)	2: 1: 7	7.3 (1.56)	1: 0: 15
Management strategies—general (*n* = 4)	6.9 (1.87)	0: 0: 2	5.9 (1.52)	0: 0: 2	7.1 (1.80)	0: 0: 2
Management strategies—specific treatments (*n* = 10)	6.0 (2.32)	0: 0: 0	4.6 (2.6)	1: 0: 0	6.3 (2.18)	0: 0: 1
General advice and mindset (*n* = 22)	7.5 (1.68)	0: 0: 19	6.2 (1.95)	0: 0: 5	7.8 (1.35)	0: 0: 19

**TABLE 5:** The concepts that are essential to include in any educational material for people with CRPS and HCP who are on the journey with them. Columns represent percentage agreement as measured on a 9‐point Likert scale. M = mean of group agreement rating, SD = standard deviation of group agreement rating, 1–3=’not important’, 4–6=’important’, 7–9=’very important’

No.		Item	Combined participant groups (HCP + CRPS)					HCP group					CRPS group				
			% agreement					% agreement					% agreement				
			M	SD	1–3	4–6	7–9	M	SD	1–3	4–6	7–9	M	SD	1–3	4–6	7–9
1.	Diagnosis	Provide details of basic signs and symptoms	7.8	1.38	0	17	83	7.8	0.96	0	0	100	7.8	1.47	0	20	80
2.	Prognosis	Describe CRPS as a long‐term disease with slow progress that often remains	7.3	1.89	8	8	83	7.5	0.57	0	0	100	7.2	2.06	10	10	80
3.		Explain that persistent CRPS may be harder to diagnose and treat compared to early CRPS	7.3	2.24	8	13	79	8.3	0.5	0	0	100	7.1	2.40	10	15	75
4.	Mechanism—‘not all in the head’	Detail that those living with CRPS will often experience effects of their pain on their mood	7.5	1.44	0	21	79	8.3	0.5	0	0	100	7.3	1.52	0	25	75
5.		Describe that although the original trauma has fully healed, the nociceptors stay activated	7.8	1.09	0	13	88	7.5	1.29	0	25	75	7.9	1.07	0	10	90
6.		Explain that pain ceases to serve a protective purpose when it persists	7.3	1.43	4	8	88	7.3	0.5	0	0	100	7.4	1.56	5	10	85
7.	Mechanism—causes	Advise that there is nothing that the person with CRPS has done that has caused the CRPS to develop, it can happen to anyone	8.0	1.52	4	8	88	6.5	2.38	25	0	75	8.4	1.13	0	10	90
8.		Explain that CRPS can be caused by very minor injuries, such as a bee sting	7.8	1.37	4	4	92	7.3	2.87	25	0	75	7.9	0.94	0	5	95
9.		Describe the role of inflammation in the acute response in chronic CRPS	7.8	1.20	0	8	92	7.8	0.95	0	0	100	7.9	1.26	0	10	90
10.	Lived experience	Explain that colour changes, swelling, excess sweating, nail and hair changes are common with CRPS	7.9	1.38	4	4	92	7.8	0.5	0	0	100	7.9	1.50	5	5	90
11.		Advise that there is currently no cure for CRPS	7.6	1.82	8	4	88	6.8	3.20	25	0	75	7.8	1.48	5	5	90
12.	Social factors/support	Advise to seek and have contact with support groups and patient organizations on CRPS	7.5	1.50	0	25	75	7	1.41	0	25	75	7.6	1.53	0	25	75
13.		Advise the importance of understanding that the people around you (family and friends) may have no clue and may be insensitive	7.6	1.41	0	21	79	7	0.81	0	25	75	7.8	1.48	0	20	80
14.		Provide advice on how to maintain employment	7.3	1.43	0	21	79	7 8	0.5	0	0	100	7.3	1.55	0	25	75
15.	Education/understanding	Provide information on things that can cause flares	7.6	1.41	0	13	88	7	2.16	0	25	75	7.8	1.25	0	10	90
16.		Provide information on how pain works and why it affects the brain and everyday life	7.6	1.42	0	10	90	6.6	1.35	0	20	80	7.8	1.34	0	8	92
17.		Describe the importance of maintaining mobility	8.2	1.15	0	10	90	7.6	1.01	0	20	80	8.4	1.13	0	8	92
18.		Describe the pathophysiology of CRPS	7.7	1.13	0	12	88	8.3	0.5	0	0	100	7.6	1.19	0	15	85
19.		Explain that new science and understanding is constantly occurring and may lead to new treatments	7.7	1.30	0	21	79	7	1.41	0	25	75	7.9	1.26	0	20	80
20.		Describe the difference between early and persistent CRPS	7.2	1.67	4	17	79	8.8	0.5	0	0	100	6.9	1.65	5	20	75
21.		Provide a definition of CRPS, incorporating the potential physical and psychological aspects	8.0	1.14	0	8	92	7.8	0.95	0	0	100	8.1	1.19	0	10	90
22.	Medications and supplements	Explain that available drugs are currently not very effective in treating CRPS	7.2	1.56	4	17	79	7.8	0.5	0	0	100	7.1	1.68	5	20	75
23.		Explain that those living with CRPS should be informed that when taking more than one medication there is the potential for drug interactions to occur	7.3	1.68	4	17	79	7	1.41	0	25	75	7.4	1.75	5	15	80
24.	Early Interventions	Encourage to begin early intervention treatments if CRPS is suspected ‐ before waiting for an official diagnosis	8.0	1.15	0	9	91	7	1.41	0	25	75	8.3	0.99	0	5	95
25.	Healthcare advice	Explain the importance of having an Exercise Physiologist in your multidisciplinary team	7.8	1.65	4	9	87	8	0.81	0	0	100	7.7	1.79	5	11	84
26.		Advise to not assume that all health professionals know how to treat CRPS	7.5	2.06	9	9	83	6.8	2.5	25	0	75	7.7	2.00	5	11	84
27.		Explain the importance of having a Physiotherapist in your multidisciplinary team	8.0	1.38	4	7	89	7.8	1.16	0	20	80	8.1	1.42	4	4	91
28.		Explain the importance of having a Psychologist in your multidisciplinary team	7.4	1.56	4	18	79	7	0.63	0	20	80	7.5	1.69	4	17	78
29.		Describe evidence‐based treatment options for CRPS	7.9	1.32	3	9	89	6.8	2.03	20	0	80	8.1	1.03	0	10	90
30.	Self‐management and advocacy	Promote Self‐management as the key to effective CRPS management	7.6	1.22	0	14	86	7	1.67	0	20	80	7.8	1.04	0	13	87
31.		Describe self‐management strategies	8.2	1.21	0	9	91	7.8	1.6	0	20	80	8.2	1.11	0	7	93
32.		Provide management strategies and how to manage the pain	7.9	1.34	0	17	83	7.8	1.16	0	20	80	8	1.36	0	17	83
33.	Management strategies—psychological	Explain the effectiveness of meditation in managing CRPS	7.4	2.13	13	4	83	6.5	2.38	25	0	75	7.6	2.09	11	5	84
34.		Explain the role of a Psychologist and the effectiveness of receiving psychological support in the management of CRPS	7.5	1.56	4	13	83	7.5	1	0	0	100	7.5	1.67	5	16	79
35.	Management strategies—Physiotherapy, Exercise and pacing	Advise to know your limits and do not go past them as it will bring on a flare	7.4	1.97	9	13	78	6.8	2.62	25	0	75	7.6	1.86	5	16	79
36.		Advise to not feel guilty that others in the house are doing more than you	7.2	2.50	13	4	83	6.3	3.59	25	0	75	7.4	2.29	11	5	84
37.		Emphasize the importance of pacing in managing CRPS	7.7	1.76	4	4	91	6.3	3.59	25	0	75	8.1	1.02	0	5	95
38.		Advise that when practicing movement, start slow and comfortably within surroundings that are familiar and safe	7.7	1.53	0	13	87	7	2.16	0	25	75	7.8	1.39	0	11	89
39.		Advise that those with CRPS need the rest of their body to be as flexible as possible	7.1	1.82	9	9	83	6.3	2.21	25	0	75	7.3	1.73	5	11	84
40.		Advise that appropriate gentle exercising of the affected limb/s can improve functioning by reducing pain sensitivity	7.5	1.30	0	15	85	7.2	0.97	0	20	80	7.6	1.36	0	14	86
41.		Advise that movement does not help	2.2	1.67	81	15	4	2.6	2.33	80	0	20	2.1	1.45	81	19	0
42.		Detail the importance of finding a Physiotherapist that can help you understand and provide support	7.2	1.40	4	12	85	6.6	1.85	20	0	80	7.4	1.21	0	14	86
43.	Management strategies—general	Summarize the importance of setting realistic goals	7.7	1.19	0	13	87	7.5	0.57	0	0	100	7.7	1.29	0	16	84
44.		Summarize the importance of looking for ways to compensate for any loss of physical mobility	7.3	1.74	4	13	83	6.8	1.25	0	25	75	7.4	1.83	5	11	84
45.	General advice and mindset	Advise that this diagnosis is not the end of the world because you are not alone	7.7	1.39	0	18	82	7	2	0	25	75	7.8	1.24	0	17	83
46.		Advise to look back regularly and remind yourself how much you have improved in order to achieve what you can do now	7.8	1.47	0	18	82	7.3	0.95	0	25	75	7.9	1.55	0	17	83
47.		Advise to look at what you can still do and not focus on what you cannot do	8.2	1.33	4	4	92	6.8	2.03	20	0	80	8.5	0.79	0	5	95
48.		Advise that CRPS is not fatal and can be treated	7.1	1.90	4	19	77	7.2	1.83	0	20	80	7.0	1.92	5	19	76

## 4 DISCUSSION

The primary aim of this study was to establish a core set of educational concepts about CRPS, using input from two participant groups (HCPs and those with CRPS), to guide future educational material. Using an e‐Delphi approach, we established a core set of 48 concepts that were independently rated as ‘very important’ by both groups. The identification of these core concepts has important implications for the development of education material about CRPS. Overall, people with CRPS rated many more concepts as ‘very important’ than the HCP group did. Many of these concepts fell under themes of ‘Lived Experience’, ‘Mindset’ and ‘Social Factors’, which highlights a need for the journey to be shared and understood between people with CRPS, their carers and social circle. In contrast, concepts rated as ‘very important’ by the HCP group fell under themes of ‘Prognosis’, ‘Diagnosis’ and ‘Mechanisms’. That these concepts more frequently reached consensus for HCPs than for people with CRPS, suggests the HCP's strong drive to understand the disorder.

### 4.1 Primary analysis

The 48 ‘essential concepts’ reflect overlap between the groups. Independently, both groups agreed that knowing about causes, prognosis, effective self‐management strategies, reaching out for social support, efforts to keep active and the understanding that CRPS symptoms can be influenced by a wide range of factors, were ‘very important’. Consensus on the importance of knowing about multidisciplinary care, with particular emphasis on the role of Physiotherapists and Psychologists, the self‐management options and understanding the likely effects of medication, were in line with published best practice guidelines (Goebel et al., 2018), recommendations and standards (Goebel et al., 2019; Goh et al., 2017). The importance of the role of Exercise Physiologists in the management of CRPS is not explicitly stated in the guidelines (Goebel et al., 2018). Perhaps the endorsement of an Exercise Physiologist in the team supports the importance of whole‐body fitness and raises the possibility that moving CRPS management away from clinical environments and into environments that are associated with wellness may afford additional benefits. Further exploration of this seems warranted. Effective pain education reached a consensus as ‘very important’ by both groups, aligning with previous suggestions that it should be incorporated into effective CRPS management (Pardo et al., 2018; Pires et al., 2016). Future educational material should endeavour to incorporate these 48 core concepts.

Some evidence exists for the importance of pain acceptance in promoting the pain‐coping behaviours for people with CRPS (Cho et al., 2013), but what effect this might have on psychological distress or other factors associated with CRPS needs further investigation. The importance of meditation has not been established in CRPS. Furthermore, social factors such as contact with support groups and patient organizations and obviating blame for the start of CRPS (it is no‐one's fault) do not currently align with standards for care (Goebel et al., 2019), but their inclusion in the 48 core concepts suggests that further research is needed to establish their importance in CRPS care.

### 4.2 Secondary analysis

Differences existed between the HCPs and people living with CRPS on the importance of various concepts. First, the HCP group reached a consensus of ‘not important’ for many more concepts than did the CRPS group. Less mainstream medical topics such as diet and alternative therapies were rated as strongly ‘not important’. This raises the possibility that HCPs are unlikely to promote strategies that lack an evidence base (such as some alternative therapies) (Maha & Shaw, 2007), or for which evidence of a clear link to the disorder is currently lacking (such as diet). In addition, the HCPs were split on their ratings of several concepts. For example, ‘mindfulness can be an effective pain management strategy’ and ‘the ability to cope with pain improves over time’ split the HCP group, with half rating these concepts as ‘not important’ and half rating them as ‘very important’. In contrast, the CRPS group were united in rating these two concepts as ‘very important’.

People with CRPS rated concepts about the lived experience as ‘very important’, for example, ‘it is not helpful to compare one case with another’, and ‘knowledge of one's own body is important—everyone is different’. No concepts, however, within this theme (Individualization) reached consensus in the HCP group. HCPs often strive for individualized patient care, yet time constraints are a common and major barrier in developing effective patient‐professional partnerships (Fu et al., 2018). Insufficient time for patient appointments due to systemic factors and healthcare policies often restricts the HCP's capacity to explore and digest the patients' experiences, therefore, impairing their ability to provide patient‐centred and individualized care (Fu et al., 2018). Changes to health care policy that enable sufficient HCP session times for CRPS patients to receive more effective, tailored management seem desirable. Additionally, future research to identify the competency, knowledge assets and clinical skills that HCPs need to engage in effective therapeutic partnerships with people with CRPS is critical.

Two‐thirds of participants with CRPS reported having the condition for 12 months or less and it is plausible that the stage of the disorder may have influenced the concepts that came to a consensus in this cohort. The themes with the highest consensus for people with CRPS were General Advice and Mindset which focussed on the individual journey and inspiring hope, and Self‐management and Advocacy which focussed on encouraging people with CRPS to research and understand the condition to ‘become your own advocate’. These concepts may reflect that CRPS is generally not well understood by HCPs (Grieve et al., 2016; Rodham et al., 2016; Rodham, Boxell, et al., 2012)—even to the point of denial of the disorder in some cases—(Bharwani et al., 2021), which often leads to uncertainty about the diagnosis and a lack of sufficient information to understand or manage the disorder (Moore et al., 2021). Future efforts made by HCPs to incorporate more messages of hope and support to those living with CRPS seem relevant based on CRPS group ratings, particularly in the early stages of diagnosis and management. Such messages would give voice to the HCP's concern about the often‐distressing lived experience of this condition. The symptoms and signs do improve in the first year for the majority of people with CRPS (Bean et al., 2016; Breivik & Stubhaug, 2015) suggest that the emphasis on what informational concepts might be most relevant may also change as the disease duration extends, and this may be a direction for future research.

### 4.3 Demographic characteristics

During recruitment, 7 of the original 16 invited advanced practitioner HCPs completed the initial survey. Our aim to reach at least 15 participants in this group was not met. Of interest, during piloting, several CRPS Taskforce HCPs reported being uncomfortable about being considered a CRPS expert because they did not see enough people with CRPS or they held a belief that their knowledge was not up to date with the current recommendations for CRPS. It is also possible that some did not take part because they felt challenged by the task of providing answers to the question from the person with CRPS ‘What would you do, if you were in my shoes?’ (Tonelli & Sullivan, 2019). The rapport between people with persistent pain and clinicians is often hampered by the difficulty that people with persistent pain have in reporting their personal experience of pain and the lack of association between what is reported, what can be seen, and what is detectable (Ashton‐James et al., 2017). These challenges undermine a shared understanding which was reflected by those in the CRPS group, who raised the importance of educating HCPs about the condition. The CRPS group also raised the importance of ‘finding someone [HCP] who is kind, to be with you through the tough times’ and an HCP who will provide ‘information on how you explain CRPS to friends and family so they understand your pain and limitations’. Furthermore, ‘information on how to advocate with medical professionals who do not listen’ was rated ‘very important’ by the CRPS group highlighting the importance of developing HCP/CRPS rapport (Ashton‐James et al., 2017).

### 4.4 Educational beliefs

Most participants felt that people with CRPS and HCPs have access to adequate educational resources on CRPS, and 85% of those in the CRPS group had educated themselves through the Internet once diagnosed with the condition. This is notable because a recent systematic review by our group (Moore et al., 2021) showed that most online resources from credible sources—at least those accessible from Australia—are not comprehensive and lack accuracy. Further, that 60% of participants with CRPS believed that they received inadequate information about CRPS from their HCP raises two possibilities: (i) that HCPs are uninformed about CRPS or (ii) that HCPs are informed about CRPS but the information they offer patients contrasts with that available online. Either scenario presents a clear and important gap in the field: accurate, comprehensive information about CRPS is required and needs to be immediately available to both consumers and HCPs.

### 4.5 Limitations

A major limitation of this study is that we did not reach our recruitment target for advanced practitioner HCP experts, and further that our recruitment strategy attracted only medical professionals, the majority in academic positions. It is likely that this limited the breadth of opinions captured in the HCP group and that there may have been more overlap on highly ranked concepts between groups if the HCP group was larger and more representative of the array of clinicians that typically work with people with CRPS. The decision to recruit HCPs from a small pool of experts, who are active in CRPS management and research and who have a working knowledge of evidence‐based practice guidelines (e.g. Goebel et al., 2018) was made to ensure we captured current, evidence‐based educational concepts. There is compelling evidence (such as the average length of time to diagnosis) to suggest that most HCPs do not have a deep understanding of CRPS or its management (Grieve et al., 2016; Rodham et al., 2016; Rodham, Boxell, et al., 2012) raising the possibility that their opinions on educational concepts may not be well informed. The generalizability of these findings, however, is limited by our recruitment strategy.

That we recruited many more CRPS than we did HCP participants may affect our results, skewing the outcomes towards the reports from people with CRPS. One consideration that may mitigate the impact of this imbalance, however, is that one would expect greater homogeneity amongst HCP experts because they gain their understanding and expertise through similar methods and sources. In contrast, CRPS experts have individual experiences—a fact that was born out in the findings, and as such will be more variable in their perspectives.

We ensured that every CRPS participant met the Budapest diagnostic symptomatic criteria (Harden et al., 2010). We were unable to use the most recent Valencia criteria because these criteria were established after we had commenced data collection (Goebel et al., 2021). That we were unable to physically assess participants meant that we could not determine criteria related to diagnostic signs, so it is possible that we included participants who would not be diagnosed with CRPS on clinical examination. We mitigated this risk by purposive recruitment through the networks of HCPs having access to patient networks. That only seven HCP experts participated, instead of our targeted 15, may mean our sample was not fully representative of experts in the field.

To obtain representative input, given the small number of people with this rare condition, international recruitment and survey e‐Delphi was necessary. Whilst enabling access to a wider sample of participants, which increases generalizability, it raises the possibility that input from people who have limited access to internet is missed, and these people may represent an under‐served cohort.

Finally, that we lodged and locked our protocol a priori and have clearly noted deviations from the original plan, is a strength of the current study. This practice is now recommended in the pain field (Lee et al., 2018) and allows the reader confidence in the transparency of our process and reporting.

## 5 CONCLUSIONS

Of 208 concepts generated, we identified 48 concepts that are essential to include in any educational material for people with CRPS and HCPs. Providing essential information is the first step to building rapport, mutual respect, and optimizing outcomes for people with CRPS and HCPs. Future investigations into the meaning and therapeutic value of the concepts that reached consensus in only one participant group are warranted. The design of future research needs to consider the opinions of a wider range of HCPs and would benefit from being undertaken with both groups involved in all stages of study design.

## AUTHOR CONTRIBUTIONS

Moore (study design, data analysis/interpretation, manuscript preparation, final approval of submitted version), Berryman (study design, data collection/analysis/interpretation, manuscript preparation, final approval of submitted version), Moseley (study design, data interpretation, manuscript preparation, final approval of submitted version), Stanton (study design, manuscript preparation, final approval of submitted version), Braithwaite (study design, data interpretation, manuscript preparation, final approval of submitted version), Bellan (survey design, manuscript preparation, final approval of submitted version).

## CONFLICT OF INTEREST

TRS has received speaker fees for lectures on pain and rehabilitation. GLM has received support from: Reality Health, ConnectHealth UK, Seqirus, Kaiser Permanente, Workers' Compensation Boards in Australia, Europe and North America, AIA Australia, Arsenal Football Club. Professional and scientific bodies have reimbursed him for travel costs related to the presentation of research on pain at scientific conferences/symposia. He has received speaker fees for lectures on pain and rehabilitation. He receives book royalties for books on pain and rehabilitation. CB has received support from ReturntoWorkSouthAustralia, South Australian Health Department and Kaiser Permanente and speaker fees for lectures on pain and rehabilitation. FAB has received speaker fees for providing lectures related to pain and blinding in clinical trials.

## Supporting information


Appendix S1
