# Supplementary material for: What do I need to know? Essential educational concepts for complex regional pain syndrome
Source: Eur J Pain. 2022 Jun 3;26(7):1481–98. doi: 10.1002/ejp.1976 (PMC9542775; doi:10.1002/ejp.1976)
Supplement: Supplementary file 1 — Appendix S1 [file EJP-26-1481-s001.docx]

**Appendices**

**TableS1** Lived Experience of participants currently living with/recovered from CRPS

| Lived Experience | Subcategories | Y:N | % of Y  (*n =* 54) | % of N  (*n =* 54) | ‘Other’ description |
| --- | --- | --- | --- | --- | --- |
| Sensory changes (Total) |  | 53:1 | 98 | 2 |  |
|  | Numbness | 53:1 | 98 | 2 |  |
|  | Pins and needles | 46:8 | 85 | 15 |  |
|  | Allodynia | 46:8 | 85 | 15 |  |
|  | Hyperalgesia | 43:11 | 80 | 20 |  |
|  | Sensitivity to warm temp | 42:12 | 78 | 22 |  |
|  | Sensitivity to cool temp | 41:13 | 76 | 24 |  |
|  | Feelings of fullness | 24:30 | 44 | 56 |  |
|  | Other | 22:32 | 41 | 59 | Burning pain/sensation (x5)  Electricity pain/sensation (x3) Cramping/ Spasms  Feeling like walking ‘on balls’  Feeling constantly cold |
| Swelling Changes (Total) |  | 49:5 | 91 | 9 |  |
|  | Appears more swollen than unaffected side | 42:12 | 78 | 22 |  |
|  | Swells when moved/used | 31:23 | 57 | 43 |  |
|  | Swells for no reason | 28:26 | 52 | 48 |  |
|  | Swells when immobilised | 21:33 | 39 | 61 |  |
|  | Feels constantly swollen | 18:36 | 33 | 67 |  |
|  | Other | 7:47 | 13 | 87 | Changes every hour Not always swollen Had initial swelling, but has since resolved (x2)  Swelling was worse in the first 5 years |
| Excess sweating |  | 38:16 | 70 | 30 |  |
|  | AL is sweatier than UAL side | 25:29 | 46 | 54 |  |
|  | AL excessively sweats when moved | 19:35 | 35 | 65 |  |
|  | AL excessively sweaty when immobilised | 9:45 | 17 | 83 |  |
|  | Other | 6:48 | 11 | 89 | Some parts of AL sweats constantly |
| Colour Changes |  | 52:2 | 96 | 4 |  |
|  | AL is a different colour/shade to UAL side | 44:10 | 81 | 19 |  |
|  | AL changes colour frequently throughout day | 38:16 | 70 | 30 |  |
|  | AL is blotchy compared to UA side | 25:29 | 46 | 54 |  |
|  | Other | 6:48 | 11 | 89 | Redness Purple/blue (x3)  The weather and barometer determines how much I’m affected  AL tends to go darker during a flare  Colour changes were worse in first couple years  Colour changes come with pain |
| Skin, Hair or Nail changes |  | 48:6 | 89 | 11 |  |
|  | Changes to nail strength/integrity | 41:13 | 76 | 24 |  |
|  | AL has a shiny appearance | 34:20 | 63 | 37 |  |
|  | Changes in skin irritability | 34:20 | 63 | 37 |  |
|  | Changes to skin strength/integrity | 28:26 | 52 | 48 |  |
|  | Changes in hair thickness | 20:34 | 37 | 63 |  |
|  | Changes in hair colour | 13:41 | 24 | 76 |  |
|  | Other | 8:46 | 85 | 15 | Skin looks drier (x4)  Faster hair growth Hair growing in new places  Wounds heal slowly Itchy  Nails grow quicker on UAL side  Slowed nail growth  Hair is longer  Limited hair growth on AL  Skin lesions (x2) Hair loss  Blisters |
| Temperature Changes |  | 53:1 | 98 | 2 |  |
|  | AL feels cooler | 47:7 | 87 | 13 |  |
|  | Changes in temperature | 36:18 | 67 | 33 |  |
|  | AL feels warmer | 22:32 | 41 | 59 |  |
|  | Other | 7:47 | 13 | 87 | One AL feels hot, the other AL feels cold (x2) Depends on surrounding environment (weather) Some affected areas go hot while others go cold  Always cold  Cold during the day and exposed to cold – hot during the night  Can change from extremely hot to cold (x2) |
| Movement/ Control changes |  | 52:2 | 96 | 4 |  |
|  | Weakness | 43:11 | 80 | 20 |  |
|  | Decreased flexibility | 42:12 | 78 | 22 |  |
|  | Cramping | 40:14 | 74 | 26 |  |
|  | Decreased ROM | 40:14 | 74 | 26 |  |
|  | Fear of moving AL | 16:38 | 30 | 70 |  |
|  | Clumsiness | 32:22 | 59 | 41 |  |
|  | AL moves involuntarily | 30:24 | 56 | 44 |  |
|  | Tremors/shakiness when moving | 29:25 | 54 | 46 |  |
|  | Tremors/shakiness when not moving | 29:25 | 54 | 46 |  |
|  | Other | 4:50 | 7 | 93 | Tremors after loud sounds  Complete loss of coordination  Unreliability whether limb will work  Unable to control  Unable to move parts of AL (fingers)  Increased pain  Dystonia |

UAL = unaffected
AL = affected limb
ROM = range of movement/motion

**TableS2**. Beliefs regarding adequate education and education received

|  | | **CRPS Group** | **Professional Group** |
| --- | --- | --- | --- |
|  | | Y:N  (n=55) | Y:N  (*n=*7) |
| Belief that those with CRPS have adequate and helpful resources available about their condition | | 34: 21 | 4: 3 |
| Belief that health professionals are up-to-date on CRPS information | | 23: 32 | 2:5 |
| **Inadequate education received by diagnostic practitioner (*n =* 33)** | | | |
| **Country currently living/Total** | **Diagnosis made by** | | |
| The Netherlands: 13/26  Canada: 14/19  Belgium: 2/2  Australia: 1/2 Germany: 1/2  United States: 1/1  Bali: 1/1 | Pain Specialist: 10  Physiotherapist: 3  Rheumatologist: 1  Specialist medical practitioner: 9  General Practitioner: 2  Psychiatrist: 1  Other: 6 | | |
| **Adequate education received by diagnostic practitioner (n – 22)** | | | |
| **Country currently living/Total** | **Diagnosis made by** | | |
| The Netherlands: 13/26  Australia: 1/2 Korea: 1/1  Canada: 5/19 Germany: 1/2  France: 1/1 | Pain Specialist: 12  Physiotherapist: 2  Rheumatologist: 2  Specialist medical practitioner: 5  Other: 1 | | |

**TableS3**. How CRPS patients educated themselves on their condition

| **Method** | **Y:N** | **% of Y  (*n =* 54)** | **% of N  (*n =* 54)** | **‘Other’ description** |
| --- | --- | --- | --- | --- |
| Google/Internet | 46:8 | 85 | 15 |  |
| Online Support Groups | 30:24 | 56 | 44 |  |
| Online Brochures | 20:34 | 37 | 63 |  |
| Brochures provided by health professionals or clinics/hospitals | 17:37 | 31 | 69 |  |
| Face-to-Face support Groups | 9:45 | 17 | 83 |  |
| Friends/family members | 7:47 | 13 | 87 |  |
| Other | 20:34 | 37 | 63 | Education by physicians (x2)  patient association (x2)  Peer-reviewed Journal Articles (x2)  YouTube (x2)  Physiotherapist  Social media (x3)  Medical library  Books (x2)  Other people with CRPS (x2) |

**tableS4:** Detailed Concept Ratings

| **No.** |  | **Item** | **Combined Expert Group Expert agreement within a 9- Point Likert Scale** | | | | | **HCP Group Expert agreement on a 9-Point Likert Scale** | | | | | **CRPS group Expert agreement on a 9-Point Likert Scale** | | | | |
| --- | --- | --- | --- | --- | --- | --- | --- | --- | --- | --- | --- | --- | --- | --- | --- | --- | --- |
|  |  |  |  | | **%** | | |  | | **%** | | |  | | **%** | | |
|  |  |  | **M** | **SD** | **1-3** | **4-6** | **7-9** | **M** | **SD** | **1-3** | **4-6** | **7-9** | **M** | **SD** | **1-3** | **4-6** | **7-9** |
|  | **Phenotype** | Describe the different phenotypes and mechanism based treatment options | 7.1 | 1.73 | 4 | 29 | 67 | 7.5 | 1.73 | 0 | 25 | 75 | 7.1 | 1.76 | 5 | 30 | 65 |
|  | **Diagnosis** | Define CRPS as a neurological condition | 6.9 | 2.17 | 8 | 21 | 71 | 5.5 | 3.69 | 25 | 25 | 50 | 7.2 | 1.73 | 5 | 20 | 75 |
|  |  | Define CRPS as an auto inflammatory disease | 7.5 | 1.62 | 0 | 25 | 75 | 7 | 2.44 | 0 | 50 | 50 | 7.6 | 1.46 | 0 | 20 | 80 |
|  |  | Provide details of the Budapest Criteria | 7.0 | 1.94 | 4 | 29 | 67 | 6.5 | 2.08 | 0 | 50 | 50 | 7.1 | 1.95 | 5 | 25 | 70 |
|  |  | Provide details of basic signs and symptoms | 7.8 | 1.38 | 0 | 17 | 83 | 7.8 | 0.96 | 0 | 0 | 100 | 7.8 | 1.47 | 0 | 20 | 80 |
|  | **Prognosis** | Provide details of average/potential remission time frames | 6.8 | 1.62 | 0 | 46 | 54 | 7.5 | 1.29 | 0 | 25 | 75 | 6.6 | 1.66 | 0 | 50 | 50 |
|  |  | Advise that persistent CRPS is unlikely to resolve quickly | 7.0 | 2.11 | 8 | 21 | 71 | 8 | 1.15 | 0 | 0 | 100 | 6.8 | 2.21 | 10 | 25 | 65 |
|  |  | Describe CRPS as a long-term disease with slow progress that often remains | 7.3 | 1.89 | 8 | 8 | 83 | 7.5 | 0.57 | 0 | 0 | 100 | 7.2 | 2.06 | 10 | 10 | 80 |
|  |  | Explain that CRPS does not have different stages | 5.0 | 2.68 | 33 | 29 | 38 | 5.3 | 1.70 | 25 | 50 | 25 | 5 | 2.86 | 35 | 25 | 40 |
|  |  | Explain that some individuals who live with persistent CRPS and have not received adequate/early treatment may present similarly to newly diagnosed individuals | 6.3 | 2.29 | 25 | 8 | 67 | 6.5 | 2.38 | 25 | 0 | 75 | 6.3 | 2.33 | 25 | 10 | 65 |
|  |  | Explain that persistent CRPS may be harder to diagnose and treat compared to early CRPS | 7.3 | 2.24 | 8 | 13 | 79 | 8.3 | 0.5 | 0 | 0 | 100 | 7.1 | 2.40 | 10 | 15 | 75 |
|  |  | Advise that early CRPS generally has a good prognosis | 7.5 | 1.68 | 3 | 21 | 76 | 7.6 | 0.8 | 0 | 0 | 100 | 7.5 | 1.80 | 4 | 25 | 71 |
|  |  | Describe the prognosis of CRPS | 7.5 | 1.49 | 0 | 20 | 80 | 6.8 | 1.93 | 0 | 40 | 60 | 7.5 | 1.38 | 0 | 17 | 83 |
|  | **Mechanism – ‘not all in the head’** | Detail that those living with CRPS will often experience effects of their pain on their mood | 7.5 | 1.44 | 0 | 21 | 79 | 8.3 | 0.5 | 0 | 0 | 100 | 7.3 | 1.52 | 0 | 25 | 75 |
|  |  | Describe that persistent CRPS is thought to be due to trauma triggering the nociceptors, and the activated nociceptors contribute to the ongoing active biological disease progress | 6.9 | 1.75 | 8 | 25 | 67 | 6.3 | 2.21 | 25 | 0 | 75 | 7 | 1.68 | 5 | 30 | 65 |
|  |  | Describe that although the original trauma has fully healed, the nociceptors stay activated | 7.8 | 1.09 | 0 | 13 | 88 | 7.5 | 1.29 | 0 | 25 | 75 | 7.9 | 1.07 | 0 | 10 | 90 |
|  |  | Explain that pain ceases to serve a protective purpose when it persists | 7.3 | 1.43 | 4 | 8 | 88 | 7.3 | 0.5 | 0 | 0 | 100 | 7.4 | 1.56 | 5 | 10 | 85 |
|  |  | Explain that CRPS is not imagined or 'just in the brain' | 7.8 | 1.78 | 3 | 15 | 82 | 6.2 | 2.4 | 20 | 20 | 60 | 8.1 | 1.46 | 0 | 11 | 89 |
|  |  | Explain that CRPS is not in the head, the pain is real | 8.2 | 1.08 | 0 | 9 | 91 | 7.2 | 1.16 | 0 | 40 | 60 | 8.4 | 0.94 | 0 | 4 | 96 |
|  | **Mechanism – causes** | Advise that there is no known cause of CRPS | 6.8 | 2.13 | 13 | 17 | 71 | 6 | 2.70 | 25 | 0 | 75 | 6.9 | 2.03 | 10 | 20 | 70 |
|  |  | Advise that there is nothing that the person with CRPS has done that has caused the CRPS to develop, it can happen to anyone | 8.0 | 1.52 | 4 | 8 | 88 | 6.5 | 2.38 | 25 | 0 | 75 | 8.4 | 1.13 | 0 | 10 | 90 |
|  |  | Explain that CRPS can be caused by very minor injuries, such as a bee sting | 7.8 | 1.37 | 4 | 4 | 92 | 7.3 | 2.87 | 25 | 0 | 75 | 7.9 | 0.94 | 0 | 5 | 95 |
|  |  | Describe the role of inflammation in the acute response in chronic CRPS | 7.8 | 1.20 | 0 | 8 | 92 | 7.8 | 0.95 | 0 | 0 | 100 | 7.9 | 1.26 | 0 | 10 | 90 |
|  |  | Explain the importance of including mental health issues such as PTSD and anxiety in CRPS research studies and clinical investigations | 6.9 | 1.90 | 8 | 25 | 67 | 6.5 | 2.38 | 25 | 0 | 75 | 6.5 | 1.84 | 5 | 30 | 65 |
|  |  | Explain that CRPS is an exaggerated response to an accident, injury, trauma or surgery | 7.2 | 1.60 | 3 | 22 | 75 | 7.8 | 0.74 | 0 | 0 | 100 | 7.1 | 1.70 | 4 | 26 | 70 |
|  |  | Explain that when nociceptors are activated, they secrete substances into the periphery, which may contribute to swelling, colour and temperature changes | 7.0 | 1.79 | 6 | 16 | 78 | 5.8 | 1.93 | 20 | 20 | 60 | 7.2 | 1.66 | 4 | 15 | 81 |
|  |  | Describe the role of maladaptive neuroplasticity in the acute response in chronic CRPS | 7.3 | 1.69 | 3 | 19 | 78 | 7 | 1.41 | 0 | 40 | 60 | 7.4 | 1.72 | 4 | 15 | 81 |
|  |  | Provide information on causes of CRPS | 7.3 | 1.73 | 0 | 29 | 71 | 7.5 | 1.29 | 0 | 25 | 75 | 7.3 | 1.83 | 0 | 30 | 70 |
|  |  | Explain the neuropathic component of CRPS | 7.3 | 1.71 | 4 | 17 | 79 | 6.8 | 2.22 | 0 | 50 | 50 | 7.4 | 1.63 | 5 | 10 | 85 |
|  | **Lived experience** | Explain that colour changes, swelling, excess sweating, nail and hair changes are common with CRPS | 7.9 | 1.38 | 4 | 4 | 92 | 7.8 | 0.5 | 0 | 0 | 100 | 7.9 | 1.50 | 5 | 5 | 90 |
|  |  | Advise that it is not helpful to compare one CRPS case with another | 7.3 | 2.01 | 8 | 13 | 79 | 6 | 2.16 | 25 | 25 | 50 | 7.6 | 1.93 | 5 | 10 | 85 |
|  |  | Advise that in the affected CRPS limb, there is not always decreased mobility or swelling in the entire limb | 7.0 | 2.04 | 8 | 29 | 63 | 6 | 2.44 | 25 | 50 | 25 | 7.2 | 1.96 | 5 | 25 | 70 |
|  |  | Advise that CRPS has the ability to spread to other body parts | 7.5 | 1.93 | 4 | 17 | 79 | 5.8 | 2.75 | 25 | 25 | 50 | 7.8 | 1.60 | 0 | 15 | 85 |
|  |  | Explain that colour changes in the affected limb can be minor and are not always extreme | 7.2 | 1.86 | 4 | 33 | 63 | 5.8 | 2.06 | 25 | 50 | 25 | 7.5 | 1.73 | 0 | 30 | 70 |
|  |  | Advise that pressure changes (e.g. firm versus soft touch) to the affected limb can cause flares | 7.4 | 1.74 | 4 | 25 | 71 | 5.8 | 2.06 | 25 | 50 | 25 | 7.7 | 1.52 | 0 | 20 | 80 |
|  |  | Advise that temperature changes to the affected limb can cause flares | 7.5 | 1.69 | 4 | 21 | 75 | 5.8 | 2.06 | 25 | 50 | 25 | 7.9 | 1.42 | 0 | 15 | 85 |
|  |  | Advise that there is currently no cure for CRPS | 7.6 | 1.82 | 8 | 4 | 88 | 6.8 | 3.20 | 25 | 0 | 75 | 7.8 | 1.48 | 5 | 5 | 90 |
|  |  | Advise that with the wrong treatment, CRPS may worsen over time | 7.2 | 2.20 | 8 | 17 | 75 | 5.5 | 3.10 | 25 | 25 | 50 | 7.5 | 1.90 | 5 | 15 | 80 |
|  |  | Provide strategies for maintaining coenaesthesis (general awareness of one's own body) | 7.2 | 1.61 | 4 | 25 | 71 | 6 | 2.82 | 25 | 25 | 50 | 7.4 | 1.23 | 0 | 25 | 75 |
|  |  | Explain that those living with CRPS often experience body perception disturbances in their affected limb/s and feel that their limb does not belong to them or feels 'weird' | 7.4 | 1.81 | 8 | 25 | 67 | 6.5 | 2.64 | 25 | 25 | 50 | 7.6 | 1.63 | 5 | 25 | 70 |
|  |  | Explain that the experience of feeling that their limb does not belong to those living with CRPS can be very distressing | 6.6 | 2.57 | 13 | 25 | 63 | 7.8 | 1.5 | 0 | 25 | 75 | 6.4 | 2.70 | 15 | 25 | 60 |
|  |  | Advise that those living with CRPS often show signs of disuse in their affected limb/s | 7.0 | 1.97 | 8 | 25 | 67 | 7.5 | 1.29 | 0 | 25 | 75 | 6.9 | 2.08 | 10 | 25 | 65 |
|  |  | Explain that swelling, colour and temperature changes tend to eventually stop in persistent CRPS | 6.7 | 2.57 | 21 | 13 | 67 | 7.5 | 1.29 | 0 | 25 | 75 | 6.5 | 2.74 | 25 | 10 | 65 |
|  |  | Explain that the ability to cope with the pain improves with time | 7.0 | 2.42 | 17 | 4 | 79 | 5.3 | 3.30 | 50 | 0 | 50 | 7.4 | 2.13 | 10 | 5 | 85 |
|  |  | Advise that CRPS symptoms and pain can change daily or minute to minute | 7.6 | 1.69 | 6 | 10 | 84 | 4.8 | 1.93 | 40 | 40 | 20 | 8.1 | 0.92 | 0 | 4 | 96 |
|  |  | Advise that CRPS symptoms and pain can change along with your stress levels | 7.6 | 1.71 | 6 | 10 | 84 | 4.8 | 2.13 | 40 | 40 | 20 | 8.1 | 0.87 | 0 | 4 | 96 |
|  |  | Advise that CRPS symptoms and pain can change along with the changes in weather | 7.4 | 1.89 | 6 | 13 | 81 | 4.6 | 1.49 | 20 | 80 | 0 | 8 | 1.41 | 4 | 0 | 96 |
|  |  | Advise that CRPS symptoms and pain can change for no reason at all | 7.6 | 1.26 | 0 | 16 | 84 | 5.8 | 0.97 | 0 | 60 | 40 | 7.9 | 0.97 | 0 | 8 | 92 |
|  |  | Explain that everyone's symptoms and experience with CRPS is different | 7.6 | 1.86 | 6 | 13 | 81 | 4.4 | 1.85 | 40 | 40 | 20 | 8.2 | 1.04 | 0 | 8 | 92 |
|  | **Social factors/support** | Advise that those with CRPS may lose friends, the ones you need will stay | 6.1 | 2.42 | 17 | 33 | 50 | 4.5 | 3.31 | 50 | 25 | 25 | 6.5 | 2.16 | 10 | 35 | 55 |
|  |  | Explain the importance of reaching out to others and talking to other CRPS patients | 7.3 | 1.99 | 4 | 29 | 67 | 5.8 | 2.5 | 25 | 50 | 25 | 7.7 | 1.78 | 0 | 25 | 75 |
|  |  | Advise to seek and have contact with support groups and patient organisations on CRPS | 7.5 | 1.50 | 0 | 25 | 75 | 7 | 1.41 | 0 | 25 | 75 | 7.6 | 1.53 | 0 | 25 | 75 |
|  |  | Advise the importance of understanding that the people around you (family and friends) may have no clue and may be insensitive | 7.6 | 1.41 | 0 | 21 | 79 | 7 | 0.81 | 0 | 25 | 75 | 7.8 | 1.48 | 0 | 20 | 80 |
|  |  | Advise on the importance of helping others with CRPS by giving your support | 7.0 | 1.83 | 4 | 33 | 63 | 5.5 | 2.08 | 25 | 50 | 25 | 7.4 | 1.66 | 0 | 30 | 70 |
|  |  | Encourage to speak openly to family members and tell them CRPS is not going to go away, although it may go into remission | 7.5 | 1.67 | 4 | 21 | 75 | 6.8 | 1.70 | 0 | 50 | 50 | 7.6 | 1.66 | 5 | 15 | 80 |
|  |  | Advise that family support is a key to maintaining mental health | 7.5 | 1.74 | 4 | 13 | 83 | 6.5 | 1.29 | 0 | 50 | 50 | 7.7 | 1.78 | 5 | 5 | 90 |
|  |  | Explain the importance of helpful support networks in managing CRPS | 7.6 | 1.38 | 0 | 21 | 79 | 6.8 | 1.5 | 0 | 50 | 50 | 7.8 | 1.33 | 0 | 15 | 85 |
|  |  | Encourage to seek a kind support person to be there to help with the tough times | 7.4 | 1.64 | 4 | 17 | 79 | 5.5 | 2.08 | 25 | 50 | 25 | 7.8 | 1.28 | 0 | 10 | 90 |
|  |  | Provide information on how to explain CRPS to your friends and family so they understand your pain and limitations | 7.9 | 1.32 | 0 | 13 | 87 | 5.8 | 1.16 | 0 | 60 | 40 | 8.4 | 0.84 | 0 | 4 | 96 |
|  |  | Explain that it is normal to go through the stages of grief for the losses you may experience with CRPS | 7.5 | 1.78 | 3 | 20 | 77 | 4.6 | 1.49 | 20 | 80 | 0 | 8.0 | 1.16 | 0 | 8 | 92 |
|  |  | Provide details of support networks and CRPS associations | 7.3 | 1.67 | 0 | 29 | 71 | 6.8 | 1.25 | 0 | 25 | 75 | 7.4 | 1.75 | 0 | 30 | 70 |
|  |  | Provide advice on how to maintain employment | 7.3 | 1.43 | 0 | 21 | 79 | 7.8 | 0.5 | 0 | 0 | 100 | 7.3 | 1.55 | 0 | 25 | 75 |
|  | **Education/ understanding** | Provide information and support networks to assist with managing suicidal ideation | 7.3 | 1.78 | 4 | 17 | 79 | 6.3 | 2.36 | 25 | 25 | 50 | 7.5 | 1.63 | 0 | 15 | 85 |
|  |  | Encourage to check multiple CRPS sites and cross reference for accurate information | 7.1 | 2.08 | 8 | 29 | 63 | 5.8 | 2.5 | 25 | 50 | 25 | 7.4 | 1.95 | 5 | 25 | 70 |
|  |  | Encourage CRPS patients to provide information flyers to their doctors/health professionals to help educate them on the condition | 7.3 | 1.85 | 4 | 21 | 75 | 5.3 | 2.62 | 25 | 50 | 25 | 7.7 | 1.42 | 0 | 15 | 85 |
|  |  | Encourage to subscribe to a CRPS newsletter | 6.3 | 2.22 | 13 | 38 | 50 | 4.8 | 2.21 | 25 | 50 | 25 | 6.6 | 2.13 | 10 | 35 | 55 |
|  |  | Provide Information on how to advocate with medical professionals who do not listen | 7.4 | 2.04 | 8 | 17 | 75 | 4.8 | 3.09 | 50 | 25 | 25 | 7.9 | 1.31 | 0 | 15 | 85 |
|  |  | Advise to go to a university hospital when seeking treatment | 5.7 | 2.35 | 17 | 42 | 42 | 4.5 | 1.73 | 25 | 50 | 25 | 5.9 | 2.42 | 15 | 40 | 45 |
|  |  | Provide information on things that can cause flares | 7.6 | 1.41 | 0 | 13 | 88 | 7 | 2.16 | 0 | 25 | 75 | 7.8 | 1.25 | 0 | 10 | 90 |
|  |  | Provide information on how pain works and why it affects the brain and everyday life | 7.6 | 1.42 | 0 | 10 | 90 | 6.6 | 1.35 | 0 | 20 | 80 | 7.8 | 1.34 | 0 | 8 | 92 |
|  |  | Provide resources and information if those with CRPS feel that they are not getting better | 7.6 | 1.60 | 0 | 20 | 80 | 6 | 1.67 | 0 | 40 | 60 | 7.9 | 1.38 | 0 | 16 | 84 |
|  |  | Encourage to check multiple sites and cross reference for accurate information | 6.3 | 2.15 | 0 | 20 | 80 | 4 | 1.41 | 40 | 60 | 0 | 6.8 | 1.95 | 4 | 40 | 56 |
|  |  | Describe the importance of maintaining mobility | 8.2 | 1.15 | 0 | 10 | 90 | 7.6 | 1.01 | 0 | 20 | 80 | 8.4 | 1.13 | 0 | 8 | 92 |
|  |  | Provide Information about the social/emotional outcomes of living with this disease | 7.6 | 1.42 | 0 | 23 | 77 | 6.2 | 0.97 | 0 | 40 | 60 | 7.9 | 1.32 | 0 | 20 | 80 |
|  |  | Describe the pathophysiology of CRPS | 7.7 | 1.13 | 0 | 12 | 88 | 8.3 | 0.5 | 0 | 0 | 100 | 7.6 | 1.19 | 0 | 15 | 85 |
|  |  | Detail common myths and unhelpful beliefs about CRPS | 6.7 | 2.44 | 13 | 21 | 67 | 6.5 | 2.08 | 0 | 50 | 50 | 6.7 | 2.55 | 15 | 15 | 70 |
|  |  | Explain that new science and understanding is constantly occurring and may lead to new treatments | 7.7 | 1.30 | 0 | 21 | 79 | 7 | 1.41 | 0 | 25 | 75 | 7.9 | 1.26 | 0 | 20 | 80 |
|  |  | Describe the difference between early and persistent CRPS | 7.2 | 1.67 | 4 | 17 | 79 | 8.8 | 0.5 | 0 | 0 | 100 | 6.9 | 1.65 | 5 | 20 | 75 |
|  |  | Provide a definition of CRPS, incorporating the potential physical and psychological aspects | 8.0 | 1.14 | 0 | 8 | 92 | 7.8 | 0.95 | 0 | 0 | 100 | 8.1 | 1.19 | 0 | 10 | 90 |
|  | **Medications and Supplements** | Summarise the effectiveness of Dorsal Root Ganglion Stimulator Systems in managing CRPS pain | 6.7 | 2.06 | 8 | 29 | 63 | 5.8 | 2.62 | 25 | 25 | 50 | 6.9 | 1.95 | 5 | 30 | 65 |
|  |  | Summarise the effectiveness for using Lidocaine Cream in managing CRPS pain | 5.7 | 2.26 | 21 | 33 | 46 | 4.5 | 2.38 | 50 | 25 | 25 | 5.9 | 2.21 | 15 | 35 | 50 |
|  |  | Summarise the effectiveness of EMLA Cream in managing CRPS pain | 5.7 | 2.28 | 21 | 33 | 46 | 4.5 | 2.38 | 50 | 25 | 25 | 5.9 | 2.24 | 15 | 35 | 50 |
|  |  | Summarise the effectiveness of using nerve blocks before surgery or major event | 6.5 | 1.91 | 8 | 29 | 63 | 5 | 2.44 | 50 | 25 | 25 | 6.8 | 1.71 | 0 | 30 | 70 |
|  |  | Summarise the effectiveness of IV Lidocaine before surgery or major event | 6.3 | 2.12 | 13 | 33 | 54 | 4.3 | 1.5 | 50 | 50 | 0 | 6.7 | 2.00 | 5 | 30 | 65 |
|  |  | Summarise the effectiveness of Ketamine in managing CRPS pain | 7.1 | 1.77 | 4 | 29 | 67 | 7 | 1.82 | 0 | 50 | 50 | 7.1 | 1.80 | 5 | 25 | 70 |
|  |  | Advise that your treating doctor will determine the best balance of medications to be on. It is okay to be on multiple medications | 7.1 | 2.09 | 4 | 25 | 71 | 7 | 1.82 | 0 | 50 | 50 | 7.2 | 2.18 | 5 | 20 | 75 |
|  |  | Explain that those with CRPS will be worse off or bedridden if they don't take multiple medications | 4.0 | 2.80 | 58 | 13 | 29 | 4.5 | 2.64 | 50 | 25 | 25 | 3.9 | 2.88 | 60 | 10 | 30 |
|  |  | Discuss Vitamin C in the management of CRPS | 7.2 | 1.81 | 4 | 25 | 71 | 6.3 | 2.5 | 25 | 25 | 50 | 7.4 | 1.66 | 0 | 25 | 75 |
|  |  | Discuss magnesium in the management of CRPS | 6.3 | 1.99 | 8 | 46 | 46 | 5 | 2.16 | 25 | 50 | 25 | 6.6 | 1.90 | 5 | 45 | 50 |
|  |  | Advise on no smoking, nicotine reduces blood flow which can make CRPS worse | 7.6 | 1.79 | 4 | 17 | 79 | 6.5 | 1.91 | 0 | 50 | 50 | 7.8 | 1.73 | 5 | 10 | 85 |
|  |  | Explain that available drugs are currently not very effective in treating CRPS | 7.2 | 1.56 | 4 | 17 | 79 | 7.8 | 0.5 | 0 | 0 | 100 | 7.1 | 1.68 | 5 | 20 | 75 |
|  |  | Explain that those living with CRPS should be informed that when taking more than one medication there is the potential for drug interactions to occur | 7.3 | 1.68 | 4 | 17 | 79 | 7 | 1.41 | 0 | 25 | 75 | 7.4 | 1.75 | 5 | 15 | 80 |
|  | **Individualisation** | Provide information on things that can cause flares | 7.2 | 2.12 | 10 | 17 | 73 | 4.2 | 1.93 | 40 | 60 | 0 | 7.8 | 1.55 | 4 | 8 | 88 |
|  |  | Explain that CRPS is not a one size fits all condition and neither are the treatments, medications and therapies. What works for one, may not work for another | 7.7 | 1.79 | 3 | 10 | 86 | 5.8 | 2.48 | 20 | 20 | 60 | 8.1 | 1.30 | 0 | 8 | 92 |
|  |  | Encourage to attempt multiple treatment options until you find what is right for you | 7.7 | 1.86 | 7 | 7 | 86 | 5.2 | 1.93 | 20 | 40 | 40 | 8.3 | 1.31 | 4 | 0 | 96 |
|  |  | Advise that not all medications work for everyone living with CRPS | 7.4 | 1.97 | 7 | 14 | 79 | 5.4 | 2.72 | 20 | 40 | 40 | 7.9 | 1.44 | 4 | 8 | 88 |
|  | **Alternative therapies** | Summarise the effectiveness of CBD (cannabidiol) oil in managing CRPS pain | 6.8 | 2.11 | 13 | 29 | 58 | 5.3 | 2.87 | 50 | 25 | 25 | 7.1 | 1.87 | 5 | 30 | 65 |
|  |  | Summarise the effectiveness of salty sea water as a natural remedy | 4.8 | 2.60 | 33 | 38 | 29 | 2.5 | 1.29 | 75 | 25 | 0 | 5.3 | 2.55 | 25 | 40 | 35 |
|  |  | Provide evidence based information on natural remedies/alternative therapies | 6.3 | 2.29 | 13 | 33 | 54 | 3.5 | 1.29 | 50 | 50 | 0 | 6.8 | 2.04 | 5 | 30 | 65 |
|  |  | Explain the effectiveness of Chaga Mushrooms in managing CRPS | 4.0 | 3.00 | 58 | 8 | 33 | 2.3 | 1.25 | 75 | 25 | 0 | 4.4 | 3.13 | 55 | 5 | 40 |
|  | **Hydrotherapy** | Summarise the effectiveness of hydrotherapy in managing CRPS | 6.6 | 2.28 | 13 | 25 | 63 | 4.8 | 3.30 | 50 | 0 | 50 | 7 | 1.91 | 5 | 30 | 65 |
|  |  | Explain that hydrotherapy in warm water provides a sense of safety from injury | 6.3 | 2.35 | 17 | 29 | 54 | 5 | 3.65 | 50 | 0 | 50 | 6.6 | 2.03 | 10 | 35 | 55 |
|  |  | Explain that hydrotherapy exercises helps the body cope with symptoms | 6.5 | 2.23 | 13 | 29 | 58 | 4.3 | 3.20 | 50 | 0 | 50 | 6.9 | 1.77 | 5 | 35 | 60 |
|  |  | Explain that hydrotherapy provides a sense of relaxation and improves the ability to move freely | 6.9 | 2.13 | 8 | 25 | 67 | 4.8 | 3.86 | 50 | 0 | 50 | 7.3 | 1.41 | 0 | 30 | 70 |
|  | **Stress** | Highlight the importance of managing and reducing stress levels | 7.7 | 1.63 | 4 | 11 | 86 | 6 | 2.09 | 20 | 20 | 60 | 8.1 | 1.22 | 0 | 9 | 91 |
|  | **Diet** | Summarise the effectiveness of diet in managing CRPS | 5.8 | 2.56 | 26 | 26 | 48 | 2.8 | 1.70 | 75 | 25 | 0 | 6.4 | 2.24 | 16 | 26 | 58 |
|  |  | Encourage to adopt an anti-inflammatory diet | 5.5 | 2.57 | 30 | 30 | 39 | 3 | 2.16 | 75 | 25 | 0 | 6.0 | 2.36 | 21 | 32 | 47 |
|  |  | Encourage to avoid packaged, boxed, canned, and prepared food items which contain inflammatory preservatives and additives | 5.3 | 2.65 | 30 | 30 | 39 | 3 | 2.82 | 75 | 0 | 25 | 5.8 | 2.41 | 21 | 37 | 42 |
|  |  | Advise to eat fresh, unprocessed whole foods | 6.0 | 2.43 | 17 | 39 | 43 | 3.5 | 2.08 | 50 | 50 | 0 | 6.5 | 2.19 | 11 | 37 | 53 |
|  |  | Advise to eat fruits and vegetable with lots of colours | 5.7 | 2.45 | 17 | 39 | 43 | 2.8 | 1.5 | 50 | 50 | 0 | 6.4 | 2.13 | 11 | 37 | 53 |
|  |  | Advise that diet can be helpful in managing CRPS | 5.4 | 2.54 | 30 | 30 | 39 | 2.5 | 1.29 | 75 | 25 | 0 | 6.1 | 2.29 | 21 | 32 | 47 |
|  |  | Encourage to drink at least 2l of water per day | 5.8 | 2.80 | 30 | 26 | 43 | 1.8 | 0.95 | 100 | 0 | 0 | 6.6 | 2.24 | 16 | 32 | 53 |
|  | **Early Interventions** | Advise on the early adoption of de-sensitisation therapies | 6.6 | 1.85 | 4 | 35 | 61 | 5.5 | 2.64 | 25 | 25 | 50 | 6.8 | 1.64 | 0 | 37 | 63 |
|  |  | Advise to immediately start Mirror Therapy after diagnosis | 5.9 | 2.50 | 22 | 26 | 52 | 4.3 | 2.21 | 50 | 25 | 25 | 6.3 | 2.46 | 16 | 26 | 58 |
|  |  | Advise to start the right medication immediately after diagnosis | 7.1 | 1.52 | 0 | 26 | 74 | 6.5 | 1 | 0 | 25 | 75 | 7.3 | 1.59 | 0 | 26 | 74 |
|  |  | Encourage to begin early intervention treatments if CRPS is suspected - before waiting for an official diagnosis | 8.0 | 1.15 | 0 | 9 | 91 | 7 | 1.41 | 0 | 25 | 75 | 8.3 | 0.99 | 0 | 5 | 95 |
|  |  | Detail the importance of early diagnosis | 7.5 | 1.84 | 7 | 14 | 79 | 5.6 | 1.74 | 20 | 40 | 40 | 8 | 1.56 | 4 | 9 | 87 |
|  |  | Detail the importance of early treatment | 7.6 | 1.81 | 7 | 11 | 82 | 5.6 | 1.74 | 20 | 40 | 40 | 8.0 | 1.50 | 4 | 4 | 91 |
|  |  | Advise to start Physiotherapy immediately after diagnosis | 7.5 | 1.63 | 4 | 11 | 86 | 7 | 1.41 | 0 | 40 | 60 | 7.6 | 1.66 | 4 | 4 | 91 |
|  | **Healthcare advice** | Explain the importance of having an Exercise Physiologist in your multidisciplinary team | 7.8 | 1.65 | 4 | 9 | 87 | 8 | 0.81 | 0 | 0 | 100 | 7.7 | 1.79 | 5 | 11 | 84 |
|  |  | Advise that Physiotherapy is the most important therapy in managing CRPS | 7.2 | 1.87 | 4 | 22 | 74 | 7.5 | 1.29 | 0 | 25 | 75 | 7.1 | 1.99 | 5 | 21 | 74 |
|  |  | Encourage to show respect for the medical professionals that are trying to help | 6.9 | 1.98 | 9 | 22 | 70 | 6 | 2.16 | 25 | 25 | 50 | 7.1 | 1.94 | 5 | 21 | 74 |
|  |  | Encourage CRPS patients to try and teach health professionals who are ignorant on CRPS | 6.8 | 2.17 | 13 | 26 | 61 | 4.8 | 3.30 | 50 | 0 | 50 | 7.3 | 1.66 | 5 | 32 | 63 |
|  |  | Explain that sometimes help can be more painful before it is helpful | 7.7 | 1.18 | 0 | 17 | 83 | 6.8 | 1.5 | 0 | 50 | 50 | 7.9 | 1.04 | 0 | 11 | 89 |
|  |  | Advise to not assume that all health professionals know how to treat CRPS | 7.5 | 2.06 | 9 | 9 | 83 | 6.8 | 2.5 | 25 | 0 | 75 | 7.7 | 2.00 | 5 | 11 | 84 |
|  |  | Encourage to gather a medical support team that understands CRPS and who you are happy with | 8 | 1.28 | 0 | 11 | 89 | 6 | 1.09 | 0 | 60 | 40 | 8.4 | 0.82 | 0 | 0 | 100 |
|  |  | Explain the importance of having a Pain Specialist in your multidisciplinary team | 7.9 | 1.30 | 0 | 14 | 86 | 6.8 | 0.74 | 0 | 40 | 60 | 8.1 | 1.27 | 0 | 9 | 91 |
|  |  | Explain the importance of having a Physiotherapist in your multidisciplinary team | 8.0 | 1.38 | 4 | 7 | 89 | 7.8 | 1.16 | 0 | 20 | 80 | 8.1 | 1.42 | 4 | 4 | 91 |
|  |  | Explain the importance of having a Psychologist in your multidisciplinary team | 7.4 | 1.56 | 4 | 18 | 79 | 7 | 0.63 | 0 | 20 | 80 | 7.5 | 1.69 | 4 | 17 | 78 |
|  |  | Advise to always be honest with your health professional | 7.9 | 1.95 | 4 | 14 | 82 | 5.6 | 2.87 | 20 | 40 | 40 | 8.4 | 1.17 | 0 | 9 | 91 |
|  |  | Encourage to not downplay your symptoms in front of your doctor because he/she needs to know everything you're experiencing to treat you properly. Your plan of care can only be a collaborative effort if your doctor knows your situation | 7.7 | 1.76 | 7 | 7 | 86 | 5 | 2.09 | 40 | 20 | 40 | 8.3 | 0.91 | 0 | 4 | 96 |
|  |  | Describe evidence-based treatment options for CRPS | 7.9 | 1.32 | 3 | 9 | 89 | 6.8 | 2.03 | 20 | 0 | 80 | 8.1 | 1.03 | 0 | 10 | 90 |
|  | **Self-management and advocacy** | Encourage those living with CRPS to track their pain and symptoms | 6.9 | 2.01 | 4 | 35 | 61 | 6.3 | 2.06 | 0 | 75 | 25 | 7 | 2.02 | 5 | 26 | 68 |
|  |  | Advise that CRPS patients can seek the assistance of Social Workers or health advocates if they believe they have been treated unfairly by medical staff | 6.3 | 2.44 | 17 | 26 | 57 | 5.3 | 2.87 | 50 | 25 | 25 | 6.5 | 2.36 | 11 | 26 | 63 |
|  |  | Advise that Social Workers can assist in seeking practitioners or services | 6.2 | 2.19 | 13 | 35 | 52 | 5.5 | 3 | 50 | 0 | 50 | 6.4 | 2.06 | 5 | 42 | 53 |
|  |  | Encourage to be diligent and prepared for medical appointments | 7.3 | 1.66 | 0 | 26 | 74 | 6.8 | 2.06 | 0 | 25 | 75 | 7.4 | 1.60 | 0 | 26 | 74 |
|  |  | Advise on the effectiveness of taking a confidant along to medical appointments to help remember what was discussed | 7.4 | 1.80 | 9 | 4 | 87 | 5.5 | 3 | 50 | 0 | 50 | 7.8 | 1.22 | 0 | 5 | 95 |
|  |  | Promote Self-management as the key to effective CRPS management | 7.6 | 1.22 | 0 | 14 | 86 | 7 | 1.67 | 0 | 20 | 80 | 7.8 | 1.04 | 0 | 13 | 87 |
|  |  | Explain the importance of incorporating self-management strategies; because medical treatments such as nerve blocks, medications and infusions are not a cure and can only get you so far | 7.6 | 1.41 | 0 | 25 | 75 | 6.6 | 1.85 | 0 | 40 | 60 | 7.8 | 1.19 | 0 | 22 | 78 |
|  |  | Explain the importance of being your own advocate in seeking to receive effective health care | 7.7 | 1.90 | 11 | 0 | 89 | 5.4 | 2.41 | 40 | 0 | 60 | 8.2 | 1.29 | 4 | 0 | 96 |
|  |  | Encourage those living with CRPS to research and understand the condition | 7.6 | 1.94 | 4 | 11 | 86 | 5 | 2.44 | 20 | 40 | 40 | 8.2 | 1.21 | 0 | 4 | 96 |
|  |  | Describe self-management strategies | 8.2 | 1.21 | 0 | 9 | 91 | 7.8 | 1.6 | 0 | 20 | 80 | 8.2 | 1.11 | 0 | 7 | 93 |
|  |  | Provide management strategies and how to manage the pain | 7.9 | 1.34 | 0 | 17 | 83 | 7.8 | 1.16 | 0 | 20 | 80 | 8 | 1.36 | 0 | 17 | 83 |
|  | **Management strategies - psychological** | Explain the importance of good sleep hygiene | 8.1 | 1.12 | 0 | 9 | 91 | 6.8 | 1.70 | 0 | 50 | 50 | 8.4 | 0.76 | 0 | 0 | 100 |
|  |  | Encourage to get as much rest and sleep as possible. Try to get at least 8 hours of sleep each night | 7.9 | 1.53 | 4 | 4 | 91 | 6 | 2.58 | 25 | 25 | 50 | 8.3 | 0.88 | 0 | 0 | 100 |
|  |  | Explain the effectiveness of meditation in managing CRPS | 7.4 | 2.13 | 13 | 4 | 83 | 6.5 | 2.38 | 25 | 0 | 75 | 7.6 | 2.09 | 11 | 5 | 84 |
|  |  | Encourage to seek the advice of a Pain Psychologist and work to address any psychosocial issues surrounding the pain condition | 7.4 | 1.75 | 4 | 17 | 78 | 7.8 | 0.5 | 0 | 0 | 100 | 7.4 | 1.92 | 5 | 21 | 74 |
|  |  | Explain the role of a Psychologist and the effectiveness of receiving psychological support in the management of CRPS | 7.5 | 1.56 | 4 | 13 | 83 | 7.5 | 1 | 0 | 0 | 100 | 7.5 | 1.67 | 5 | 16 | 79 |
|  |  | Explain that anger can make CRPS pain worse | 7.4 | 2.04 | 9 | 13 | 78 | 6 | 2.44 | 25 | 50 | 25 | 7.7 | 1.88 | 5 | 5 | 89 |
|  |  | Explain the importance of Mindfulness Based Stress Reduction in the management of CRPS | 7.3 | 2.10 | 9 | 13 | 78 | 6 | 2.94 | 25 | 25 | 50 | 7.6 | 1.86 | 5 | 11 | 84 |
|  |  | Explain that Mindfulness can be an effective pain management strategy | 7.3 | 2.12 | 13 | 9 | 78 | 5.8 | 3.20 | 50 | 0 | 50 | 7.6 | 1.77 | 5 | 11 | 84 |
|  |  | Explain that Body Scanning (a Mindfulness technique) can be an effective pain management strategy | 7.0 | 2.32 | 17 | 9 | 74 | 4.8 | 2.75 | 50 | 25 | 25 | 7.5 | 1.98 | 11 | 5 | 84 |
|  | **Management strategies – Physiotherapy, Exercise and pacing** | Explain that pain or additional trauma can trigger the disease, therefore pain should be kept at a tolerable/low level | 7.3 | 1.74 | 4 | 26 | 70 | 6 | 2.58 | 25 | 25 | 50 | 7.6 | 1.46 | 0 | 26 | 74 |
|  |  | Advise that Physiotherapy treatments should avoid increasing pain | 6.0 | 2.88 | 30 | 17 | 52 | 5.3 | 3.86 | 50 | 0 | 50 | 6.2 | 2.73 | 26 | 21 | 53 |
|  |  | Advise to know your limits and don't go past them as it will bring on a flare | 7.4 | 1.97 | 9 | 13 | 78 | 6.8 | 2.62 | 25 | 0 | 75 | 7.6 | 1.86 | 5 | 16 | 79 |
|  |  | Advise that it's ok to rest and put your feet up to help the symptoms | 7.2 | 1.86 | 4 | 35 | 61 | 6.3 | 2.06 | 0 | 75 | 25 | 7.4 | 1.80 | 5 | 26 | 68 |
|  |  | Advise to not feel guilty that others in the house are doing more than you | 7.2 | 2.50 | 13 | 4 | 83 | 6.3 | 3.59 | 25 | 0 | 75 | 7.4 | 2.29 | 11 | 5 | 84 |
|  |  | Emphasise the importance of pacing in managing CRPS | 7.7 | 1.76 | 4 | 4 | 91 | 6.3 | 3.59 | 25 | 0 | 75 | 8.1 | 1.02 | 0 | 5 | 95 |
|  |  | Explain that 'no pain no gain' no longer applies to those living with CRPS | 7.1 | 2.40 | 17 | 4 | 78 | 4.3 | 3.20 | 75 | 0 | 25 | 7.7 | 1.75 | 5 | 5 | 89 |
|  |  | Advise that when practicing movement, start slow and comfortably within surroundings that are familiar and safe | 7.7 | 1.53 | 0 | 13 | 87 | 7 | 2.16 | 0 | 25 | 75 | 7.8 | 1.39 | 0 | 11 | 89 |
|  |  | Explain that the aim of Physiotherapy is to regain normal function to achieve remission within the first 12-24 months | 6.4 | 2.19 | 17 | 17 | 65 | 4.8 | 2.06 | 50 | 25 | 25 | 6.7 | 2.10 | 11 | 16 | 74 |
|  |  | Advise that those with CRPS need the rest of their body to be as flexible as possible | 7.1 | 1.82 | 9 | 9 | 83 | 6.3 | 2.21 | 25 | 0 | 75 | 7.3 | 1.73 | 5 | 11 | 84 |
|  |  | Encourage to increase bodily awareness: Your body will show signs you're overdoing it, and if you learn those signs, you can lessen future suffering | 7.7 | 1.64 | 4 | 13 | 83 | 6.5 | 2.64 | 25 | 25 | 50 | 7.9 | 1.32 | 0 | 11 | 89 |
|  |  | Advise that appropriate gentle exercising of the affected limb/s can improve functioning by reducing pain sensitivity | 7.5 | 1.30 | 0 | 15 | 85 | 7.2 | 0.97 | 0 | 20 | 80 | 7.6 | 1.36 | 0 | 14 | 86 |
|  |  | Summarise the effectiveness of the 'move it or lose it' principle | 7.5 | 1.82 | 4 | 15 | 81 | 5.8 | 2.92 | 20 | 20 | 60 | 7.9 | 1.10 | 0 | 14 | 86 |
|  |  | Advise that those living with CRPS should try to move their affected limb as much as possible | 7.4 | 1.78 | 4 | 12 | 85 | 5.2 | 2.48 | 20 | 40 | 40 | 8 | 0.97 | 0 | 5 | 95 |
|  |  | Explain that it's better to start slowly with things to see how your body reacts before you dive in too quickly and cause unnecessary pain or worsening of your condition | 7.6 | 1.49 | 0 | 19 | 81 | 6.6 | 1.62 | 0 | 40 | 60 | 7.8 | 1.35 | 0 | 14 | 86 |
|  |  | Summarise the importance of Physiotherapy/exercising daily and in small batches for recovery | 7.9 | 1.26 | 0 | 12 | 88 | 6.6 | 1.49 | 0 | 40 | 60 | 8.2 | 0.97 | 0 | 5 | 95 |
|  |  | Advise that when done at the correct pace, duration and intensity, physical therapy is doing to be your best friend and worst enemy | 7.1 | 1.96 | 8 | 15 | 77 | 5.2 | 1.6 | 20 | 40 | 40 | 7.6 | 1.72 | 5 | 10 | 86 |
|  |  | Summarise the importance of attempting to keep exercising, even when it is hard | 7.4 | 1.59 | 4 | 12 | 85 | 6.4 | 1.35 | 0 | 40 | 60 | 7.7 | 1.54 | 5 | 5 | 90 |
|  |  | Advise that movement does not help | 2.2 | 1.67 | 81 | 15 | 4 | 2.6 | 2.33 | 80 | 0 | 20 | 2.1 | 1.45 | 81 | 19 | 0 |
|  |  | Detail the importance of finding a Physiotherapist that can help you understand and provide support | 7.2 | 1.40 | 4 | 12 | 85 | 6.6 | 1.85 | 20 | 0 | 80 | 7.4 | 1.21 | 0 | 14 | 86 |
|  | **Management strategies - General** | Advise to find and start new hobbies | 6.7 | 2.20 | 13 | 22 | 65 | 3.8 | 2.36 | 50 | 25 | 25 | 7.3 | 1.63 | 5 | 21 | 74 |
|  |  | Explain the importance of using national treatment rules (Leitlinie.crps-nrw.de) | 5.9 | 2.34 | 13 | 43 | 43 | 5.5 | 1.91 | 25 | 25 | 50 | 5.9 | 2.46 | 11 | 47 | 42 |
|  |  | Summarise the importance of setting realistic goals | 7.7 | 1.19 | 0 | 13 | 87 | 7.5 | 0.57 | 0 | 0 | 100 | 7.7 | 1.29 | 0 | 16 | 84 |
|  |  | Summarise the importance of looking for ways to compensate for any loss of physical mobility | 7.3 | 1.74 | 4 | 13 | 83 | 6.8 | 1.25 | 0 | 25 | 75 | 7.4 | 1.83 | 5 | 11 | 84 |
|  | **Management strategies – specific treatments** | Summarise the effectiveness of Graded Motor Imagery in managing CRPS | 6.6 | 1.84 | 5 | 45 | 50 | 5.3 | 2.06 | 25 | 50 | 25 | 6.9 | 1.71 | 0 | 44 | 56 |
|  |  | Summarise the effectiveness of relaxation, deep breathing and meditation in managing CRPS | 7.3 | 1.67 | 5 | 23 | 73 | 5.8 | 2.21 | 25 | 25 | 50 | 7.7 | 1.37 | 0 | 22 | 78 |
|  |  | Summarise the effectiveness of Mirror Therapy in managing CRPS | 6.7 | 1.81 | 5 | 41 | 55 | 5.5 | 2.08 | 25 | 50 | 25 | 6.9 | 1.69 | 0 | 39 | 61 |
|  |  | Summarise the effectiveness of yoga in managing CRPS | 6.5 | 1.99 | 14 | 27 | 59 | 4.5 | 1.91 | 50 | 25 | 25 | 6.9 | 1.77 | 6 | 28 | 67 |
|  |  | Advise to avoid using ice in managing CRPS pain and symptoms | 5.7 | 2.95 | 36 | 14 | 50 | 3.5 | 3.10 | 75 | 0 | 25 | 6.2 | 2.77 | 28 | 17 | 56 |
|  |  | Advise to use heat/heat pads in managing CRPS pain and symptoms | 5.4 | 2.68 | 32 | 27 | 41 | 4.8 | 3.30 | 50 | 0 | 50 | 5.5 | 2.61 | 28 | 33 | 39 |
|  |  | Summarise the effectiveness of service dogs in managing CRPS | 5.1 | 2.52 | 32 | 32 | 36 | 3.5 | 2.08 | 50 | 50 | 0 | 5.4 | 2.52 | 28 | 28 | 44 |
|  |  | Explain that heat therapies (e.g. heat packs) may not be a beneficial management strategy for all individuals living with CRPS | 6.0 | 2.30 | 18 | 32 | 50 | 4.5 | 3 | 50 | 0 | 50 | 6.4 | 2.06 | 11 | 39 | 50 |
|  |  | Explain that some individuals living with CRPS benefit from the use of cold therapies (e.g. ice, cold water) to relieve swelling and inflammation | 5.7 | 2.71 | 23 | 23 | 55 | 4.5 | 3 | 50 | 0 | 50 | 5.9 | 2.66 | 17 | 28 | 56 |
|  |  | Explain that in those who have a constant/heightened awareness of body perception, strategies to maintain coenaesthesis may not be not beneficial | 4.9 | 2.69 | 36 | 27 | 36 | 4.5 | 3.10 | 50 | 25 | 25 | 5 | 2.67 | 33 | 28 | 39 |
|  | **General advice and mindset** | Encourage to not care what other people think of you | 6.5 | 2.52 | 18 | 23 | 59 | 6 | 2.16 | 25 | 25 | 50 | 6.6 | 2.63 | 17 | 22 | 61 |
|  |  | Advise to find the silver lining | 6.8 | 2.40 | 14 | 18 | 68 | 6 | 3.46 | 25 | 0 | 75 | 7 | 2.19 | 11 | 22 | 67 |
|  |  | Advise that this diagnosis is not the end of the world because you are not alone | 7.7 | 1.39 | 0 | 18 | 82 | 7 | 2 | 0 | 25 | 75 | 7.8 | 1.24 | 0 | 17 | 83 |
|  |  | Advise to do everything you want to do, don't let the CRPS control your life completely | 8.2 | 1.07 | 0 | 14 | 86 | 7 | 1.15 | 0 | 50 | 50 | 8.5 | 0.85 | 0 | 6 | 94 |
|  |  | Advise to look back regularly and remind yourself how much you have improved in order to achieve what you can do now | 7.8 | 1.47 | 0 | 18 | 82 | 7.3 | 0.95 | 0 | 25 | 75 | 7.9 | 1.55 | 0 | 17 | 83 |
|  |  | Advise to stay busy during the day | 6.2 | 2.40 | 18 | 32 | 50 | 6.3 | 1.70 | 0 | 50 | 50 | 6.2 | 2.57 | 22 | 28 | 50 |
|  |  | Encourage to accept that life is going to change | 7.4 | 1.40 | 0 | 14 | 86 | 6 | 1.82 | 0 | 50 | 50 | 7.7 | 1.13 | 0 | 6 | 94 |
|  |  | Advise that accepting your life is going to change is key to mental health and reducing pain flares | 7.5 | 1.47 | 5 | 9 | 86 | 5.8 | 2.21 | 25 | 25 | 50 | 7.3 | 0.98 | 0 | 6 | 94 |
|  |  | Advise on using a sense of humour to manage clumsiness and when experiencing terrible life events | 7.1 | 2.32 | 9 | 14 | 77 | 4.5 | 3.51 | 50 | 0 | 50 | 7.7 | 1.56 | 0 | 17 | 83 |
|  |  | Advise that If you feel like you can't go on, remember there is a whole army of CRPS warriors, and we can help you keep fighting | 7.7 | 1.59 | 5 | 14 | 82 | 6 | 2.16 | 25 | 25 | 50 | 8.1 | 1.21 | 0 | 11 | 89 |
|  |  | Advise that it is okay to look well when you have a chronic illness | 7.3 | 1.97 | 4 | 19 | 77 | 5.2 | 2.48 | 20 | 40 | 40 | 7.8 | 1.42 | 0 | 14 | 86 |
|  |  | Encourage to not be ashamed of living with CRPS | 7.8 | 1.59 | 0 | 15 | 85 | 5.8 | 1.46 | 0 | 60 | 40 | 8.2 | 1.20 | 0 | 5 | 95 |
|  |  | Encourage to not be afraid of living with CRPS | 7.5 | 1.62 | 0 | 19 | 81 | 5.8 | 1.46 | 0 | 60 | 40 | 8 | 1.34 | 0 | 10 | 90 |
|  |  | Explain the importance of self-care and being kind to yourself | 8.1 | 1.59 | 4 | 12 | 85 | 6.2 | 2.4 | 20 | 40 | 40 | 8.6 | 0.77 | 0 | 5 | 95 |
|  |  | Advise to look at what you can still do and not focus on what you can't do | 8.2 | 1.33 | 4 | 4 | 92 | 6.8 | 2.03 | 20 | 0 | 80 | 8.5 | 0.79 | 0 | 5 | 95 |
|  |  | Encourage enjoyment in the little things | 7.6 | 1.74 | 4 | 12 | 85 | 5.6 | 2.05 | 20 | 40 | 40 | 8.1 | 1.21 | 0 | 5 | 95 |
|  |  | Encourage to listen to your body and do whatever you can, but take rest if your body asks for it | 7.8 | 1.35 | 0 | 15 | 85 | 6.4 | 1.35 | 0 | 40 | 60 | 8.1 | 1.12 | 0 | 10 | 90 |
|  |  | Explain that CRPS does not define you; it's just a part of your life. You are more than your pain, more than your disability | 8.0 | 1.26 | 0 | 12 | 88 | 6.2 | 1.32 | 0 | 60 | 40 | 8.5 | 0.73 | 0 | 0 | 100 |
|  |  | Advise that CRPS is not fatal and can be treated | 7.1 | 1.90 | 4 | 19 | 77 | 7.2 | 1.83 | 0 | 20 | 80 | 7.0 | 1.92 | 5 | 19 | 76 |
|  |  | Encourage to try to see the possibilities and be happy as often as possible | 7.6 | 1.54 | 4 | 15 | 81 | 6.4 | 1.35 | 0 | 40 | 60 | 7.9 | 1.44 | 5 | 10 | 86 |
|  |  | Encourage to search for ways to enjoy life despite the challenges | 7.6 | 1.68 | 4 | 12 | 85 | 5.8 | 2.22 | 20 | 40 | 40 | 8.0 | 1.17 | 0 | 5 | 95 |
|  |  | Advise to not give up | 8.0 | 1.37 | 4 | 8 | 88 | 6.4 | 1.85 | 20 | 20 | 60 | 8.4 | 0.84 | 0 | 5 | 95 |
